# Deficiency and overexpression of *Rtl1* in the mouse cause distinct muscle abnormalities related to Temple and Kagami-Ogata syndromes

**DOI:** 10.1242/dev.185918

**Published:** 2020-09-03

**Authors:** Moe Kitazawa, Shinichiro Hayashi, Michihiro Imamura, Shin'ichi Takeda, Yumiko Oishi, Tomoko Kaneko-Ishino, Fumitoshi Ishino

**Affiliations:** 1 Department of Epigenetics, Medical Research Institute, Tokyo Medical and Dental University (TMDU), Tokyo 113-8510, Japan; 2 Department of Neuromuscular Research, National Institute of Neuroscience, National Center of Neurology and Psychiatry (NCNP), Tokyo 187-8502, Japan; 3 Department of Molecular Therapy, National Institute of Neuroscience, National Center of Neurology and Psychiatry (NCNP), Tokyo 187-8502, Japan; 4 Department of Biochemistry and Molecular Biology, Nippon Medical School, Tokyo 113-8602, Japan; 5 Faculty of Nursing, School of Medicine, Tokai University, Kanagawa 259-1193, Japan

**Keywords:** Uniqueness of fetal/neonatal muscle, Temple syndrome, Kagami-Ogata syndrome, Gene domestication, Eutherian evolution

## Abstract

Temple and Kagami-Ogata syndromes are genomic imprinting diseases caused by maternal and paternal duplication of human chromosome 14, respectively. They exhibit different postnatal muscle-related symptoms as well as prenatal placental problems. Using the mouse models for these syndromes, it has been demonstrated that retrotransposon gag like 1 [*Rtl1*, also known as paternally expressed 11 (*Peg11*)] located in the mouse orthologous imprinted region is responsible for the prenatal placental problems because it is an essential placental gene for maintenance of fetal capillary network during gestation. However, the causative imprinted gene for the postnatal muscle-related symptoms remains unknown. Here, we demonstrate that *Rtl1* also plays an important role in fetal/neonatal skeletal muscle development: its deletion and overproduction in mice lead to neonatal lethality associated with severe but distinct skeletal muscle defects, similar to those of Temple and Kagami-Ogata syndromes, respectively. Thus, it is strongly suggested that *RTL1* is the major gene responsible for the muscle defects in addition to the placental defects in these two genomic imprinting diseases. This is the first example of an LTR retrotransposon-derived gene specific to eutherians contributing to eutherian skeletal muscle development.

## Abstract (precis)

**Summary:** Rtl1 plays an important role in fetal and neonatal skeletal muscle development. Abnormal expression of human RTL1 is the major cause of the muscle symptoms observed in Temple and Kagami-Ogata syndromes.

## INTRODUCTION

Temple and Kagami-Ogata syndromes are caused by maternal and paternal disomy of chromosome 14 [Upd(14)mat and upd(14)pat], respectively. The individuals with Temple syndrome exhibit certain muscle-related symptoms, such as muscle hypotonia, feeding difficulty/poor sucking function in early childhood, in contrast to neonatal lethality due to respiratory problems associated with a bell-shaped thorax and abdominal wall hernia in the Kagami-Ogata syndrome (Kagami et al., 2005, 2015; Ogata and Kagami, 2016; Kotzot, 2004; Ioannides et al., 2014). Retrotransposon gag like 1 (*RTL1*) was first identified as *PEG11* in a *DLK1*-*DIO3* imprinted region in sheep (Charlier et al., 2001), and later was formally renamed *RTL1* as one of the genes derived from a suchi-ichi-related LTR retrotransposon in humans and mice. Human *RTL1* is a paternally expressed imprinted gene located in the same *DLK1*-*DIO3* imprinted region on human chromosome 14; its mouse ortholog *Rtl1* is on mouse distal chromosome 12 (Fig. S1). Interestingly, its mRNA is regulated by seven microRNAs (miRNAs) processed from maternally expressed *RTL1as* (*RTL1* antisense transcript) through an RNAi mechanism (Davis et al., 2005) (Fig. S2A). We have previously demonstrated that mouse *Rtl1* is one of the major genes responsible for the placental abnormalities characteristic of these two syndromes, and also that the severity of the phenotypes of Kagami-Ogata syndrome, such as a bell-shaped thorax and neonatal lethality associated with respiratory problems, is associated with the degree of overproduction of human *RTL1* (Kagami et al., 2008). Beyond the essential involvement of *Rtl1* in maintaining placental fetal capillaries, the role of *Rtl1* in the respiratory failure and other muscular problems observed in neonates remains unknown, although recent studies report that the ectopic expression of ovine *RTL1* leads to muscle hypertrophy in mice, which mimics the sheep *callipyge* phenotype (Charlier et al., 2001; Fleming-Waddell et al., 2009; Byrne et al., 2010; Xu et al., 2015; Mikovic et al., 2018).

In this study, we directly addressed this issue and extensively examined the role of *Rtl1* in fetal muscle development at both cellular and tissue levels using two mouse models: Pat-*Rtl1*Δ (loss of *Rtl1*) and Mat-*Rtl1*Δ (overproduction of *Rtl1*) mice. As described in the original paper in detail (Sekita et al., 2008), the lethal phenotypes of the Pat- and Mat-*Rtl1*Δ mice are entirely dependent on the genetic background. The first generation [C57BL/6×129/Sv (B6/129) F1] did not exhibit any lethality, but a pre- and postnatal growth retardation phenotype was observed in Pat-*Rtl1*Δ mice. Subsequently, KO mice were continuously crossed to B6 mice and the lethality became apparent after the F3 and F6 generations in the Pat-*Rtl1*Δ and Mat-*Rtl1*Δ mice, respectively. The lethal phenotype became stronger in the later generations and finally all of the F10 individuals on the B6 genetic background died. For example, in the F5 generation, one half of the Pat-*Rtl1*Δ mice exhibited mid- to late fetal lethality because of the severe placental defect of destruction of the fetal capillary network (Sekita et al., 2008; Kitazawa et al., 2017) and another half exhibited late fetal growth retardation and were born small (∼80% of WT pups). Among them, one half (a quarter in total) died within 24 h of birth and another half were viable. In the case of the Mat-*Rtl1*Δ mice, neonatal lethality was predominant after the F6 generation, always in association with placentomegaly (150% by weight compared with the wild type). The placental fetal capillaries were also severely damaged, but in a different manner from those in Pat-*Rtl1*Δ. These results are consistent with those obtained using other *Rtl1* and *miR-127* deletion mutants, although both of those exhibited milder phenotypes than in our models (Ito et al., 2015). Although both the Pat- and Mat-*Rtl1*Δ mice had severe placental problems, we think it is necessary to address the abnormalities of the neonates themselves, especially the fetal/neonatal muscle phenotypes, and then the relationship to the Temple and Kagami-Ogata syndromes. In both cases, we detected severe but distinct abnormalities in several neonatal muscles that are essential for respiration, such as the intercostal, abdominal and diaphragm muscles. This is the first demonstration that an LTR retrotransposon-derived *Rtl1* plays an important role in fetal and neonatal muscle development, strongly suggesting the crucial involvement of *RTL1* in several muscle symptoms observed in the Temple and Kagami-Ogata syndromes. We further discuss an evolutionary role of the domestication of *RTL1* in fetal/neonatal muscle development as a eutherian-specific gene from an LTR retrotransposon.

## RESULTS

### Genetic models

We used knockout (KO) mice to elucidate *Rtl1* function in muscle development, as previously reported (Sekita et al., 2008) (Figs S1 and S2). In the KO allele, most of the *Rtl1* region was removed, so six out of seven microRNAs in *Rtl1as*, including *miR-434-3p*, *miR-434-5p*, *miR-127*, *miR-433-3p*, *miR-433-5p* and *miR-431*, were also removed (Fig. S2B). The mice with the deletion upon paternal transmission exhibited a loss of *Rtl1* expression phenotype (Pat-*Rtl1*Δ and *Rtl1* KO), while the mice with maternal transmission exhibited overexpression of the paternal *Rtl1* allele because of a loss of expression of most of the maternal microRNAs (Mat-*Rtl1*Δ and *antiRtl1as* KO). As the Pat- and Mat-*Rtl1*Δ mice were obtained from different mating combinations, we employed two control wild-type mice, WT(p) and WT(m), respectively (Fig. S2C). We used Pat-*Rtl1*Δ and Mat-*Rtl1*Δ mice in the F8 and F9 generations, respectively, because most of the pups exhibited neonatal lethality.

### Temporal expression of Rtl1 in fetal and neonatal skeletal muscles

**Fig. 1.**
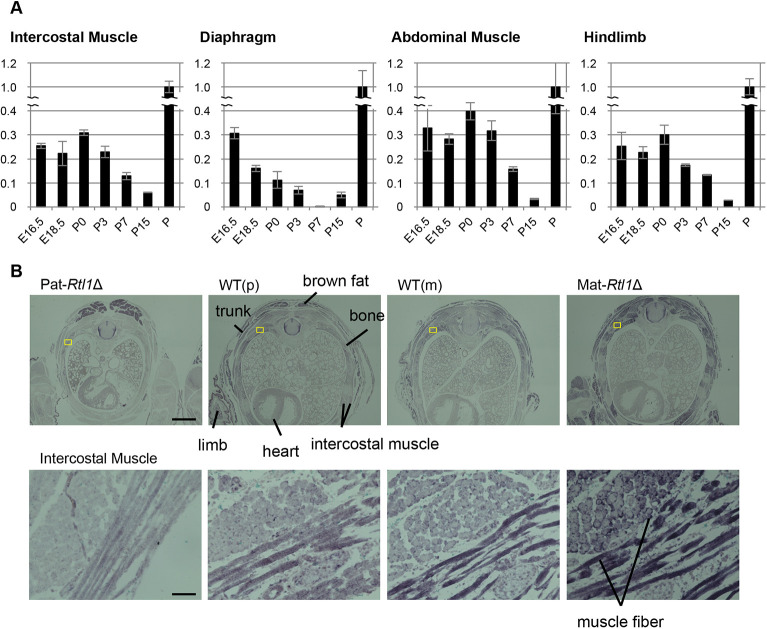
***Rtl1* mRNA expression in embryos, and in embryonal and neonatal muscles; RTL1 protein localization in neonatal intercostal muscles.** (A) Quantitative PCR results of *Rtl1* in the diaphragm, hindlimb, intercostal and abdominal muscles in E16.5 and E18.5 embryos and neonates (P0, P3, P7 and P15). Relative expression levels of *Rtl1* to *Gapdh* are shown. Placenta (P: E18.5) was used as the positive control and its *Rtl1*/*Gapdh* ratio was adjusted to 1. Data are mean±s.d. (B) Immunohistochemical staining of the RTL1 protein in neonates. A cross-sectional view of the neonates (top) and higher magnification views of the intercostal muscle (yellow boxes) are shown (bottom). The RTL1 signals (purple by BCIP/NBT staining) were observed around and along the muscle fibers. Scale bars: 1 mm (top) and 50 μm (bottom). Neonates were fixed in Super Fix.

*Rtl1* exhibits temporal muscle expression exclusively in the fetal to neonatal stages. In the embryo, its expression was detected by at least embryonic day 12.5 (E12.5), while in the skeletal muscles it was detected in E16.5 and E18.5 fetuses and neonates [postnatal day 0 (P0)]; however, its expression was gradually reduced after birth and almost entirely disappeared by P15 (Fig. 1A, Figs S3 and S4). In the heart, there was no *Rtl1* expression throughout development (Fig. 1B and Fig. S3). Immunohistochemical experiments using an anti-Rtl1 antibody clearly demonstrated the presence of RTL1 protein in E14.5 fetal myofibers (Fig. S5) and almost all of the neonatal skeletal muscles in the wild-type and Mat-*Rtl1*Δ mice, but not in the Pat-*Rtl1*Δ mice (Fig. 1B). In the western blotting experiment using E16.5 fetal diaphragm and hind limb muscle, the RTL1 protein was detected mainly as a 250 kDa band and a concomitant minor band of around 200 kDa was also detected (Fig. S6). These values are roughly consistent with the estimated molecular weight of 199 kDa from the full-length 1744 amino acid sequence of the mouse RTL1 protein. RTL1 expression was apparently higher in Mat-*Rtl1*Δ compared with the wild type. In the Pat-*Rtl1*Δ fetal diaphragm, a very small amount of the lower minor band was detected, consistent with the mRNA expression data shown in Fig. S4B, suggesting leaky expression from the maternal alleles.

### Structural abnormalities in neonatal skeletal muscles in Pat- and Mat-Rtl1Δ mice

**Fig. 2.**
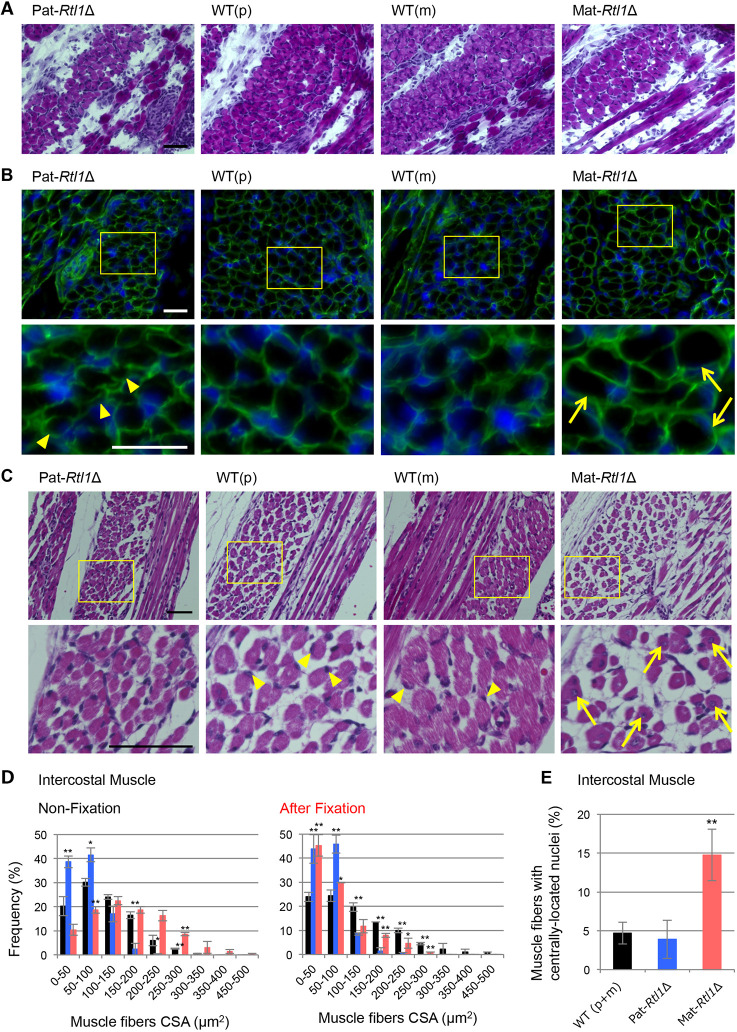
**Histological abnormalities in intercostal muscle of Pat- and Mat-*Rtl1*Δ.** (A,B) Hematoxylin and Eosin (HE) staining and immunofluorescence staining of the neonatal intercostal muscle. (A) HE staining of the neonatal intercostal muscle. (B) Co-immunostaining with laminin (green) and DAPI (blue) (top row), and higher magnification views of the intercostal muscle (yellow boxes) (bottom row). The arrowheads in the Pat-*Rtl1*Δ column indicate thinner muscle fibers and the arrows in the Mat-*Rtl1*Δ column indicate large muscle fibers. The neonates were not fixed before being embedded in OCT compound. (C) HE staining in neonate intercostal muscle (top) and higher magnification views (bottom): Pat*-Rtl1*Δ (left), wild type (middle) and Mat*-Rtl1*Δ (right). The arrowheads in the wild-type columns indicate normal nuclei and the arrows in the Mat*-Rtl1*Δ column indicate muscle fibers with centrally located nuclei. Scale bars: 50 μm. Neonates were fixed in Super Fix. (D) Distribution of the muscle fiber cross-sectional area (CSA) in wild-type (black, *n*=4), Pat-*Rtl1*Δ (blue, *n*=3) and Mat-*Rtl1*Δ (red, *n*=3) neonates [non-fixed samples (left) and fixed samples with Super Fix (right)]. (E) Proportion of muscle fibers with centrally located nuclei (arrows in C) between wild-type (black, *n*=4), Pat*-Rtl1*Δ (blue, *n*=4) and Mat*-Rtl1*Δ (red, *n*=4) neonates. Neonates were fixed in Super Fix. *P<0.05, **P<0.01 (two-tailed Student's *t*-test). Data are mean±s.d.

Hematoxylin and Eosin (HE) staining, as well as anti-laminin antibody staining revealed apparent structural abnormalities of the skeletal muscles in both the Pat-*Rtl1*Δ and Mat-*Rtl1*Δ mice. The Pat-*Rtl1*Δ mice exhibited significantly thinner muscle fibers compared with the wild-type controls (Fig. 2A,B, left), whereas the Mat-*Rtl1*Δ mice had a significantly larger muscle fiber size, as estimated by the anti-laminin antibody staining of non-fixed cryosectioned samples (Fig. 2A,B, right). Georgiades et al. also reported similar features in the diaphragm and forelimb muscles using mUPD12 and pUPD12 mice (Table 1: Georgiades et al., 2000). However, after fixation with Super Fix, the muscle fibers of the Mat-*Rtl1*Δ mice displayed severe shrinkage and became detached from the extracellular matrix (ECM) in the intercostal (Fig. 2C and Fig. S7A, Elastica van Gieson staining), diaphragm (Fig. S7B), abdominal (Fig. S7C) and limb (Fig. S7D) muscles. Therefore, a substantial muscle fiber cross-sectional area of the Mat-*Rtl1*Δ mice had shrunk as in the case of the Pat-*Rtl1*Δ mice (Fig. 2D and Fig. S7E), suggesting some unknown structural abnormality in the Mat-*Rtl1*Δ myofibers. Notably, the proportion of centronuclear muscle fibers was significantly high in the intercostal and limb muscles of the Mat-*Rtl1*Δ mice (*P*=0.000224 and *P*=0.000143, two-tailed Student's *t*-test, respectively), also suggesting defects in muscle fiber maturation (Georgiades et al., 2000; Fürst et al., 1989) (Fig. 2C,E, Fig. S7D,F). We did not observe such differences in the diaphragm or abdominal muscles (Fig. S7F).

**Table 1.**
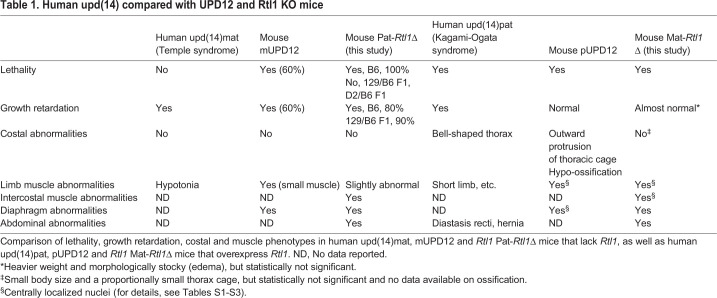
**Human upd(14) compared with UPD12 and Rtl1 KO mice**

### Localization of RTL1 protein in adjoining area of the Z-disc close to the DESMIN protein

**Fig. 3.**
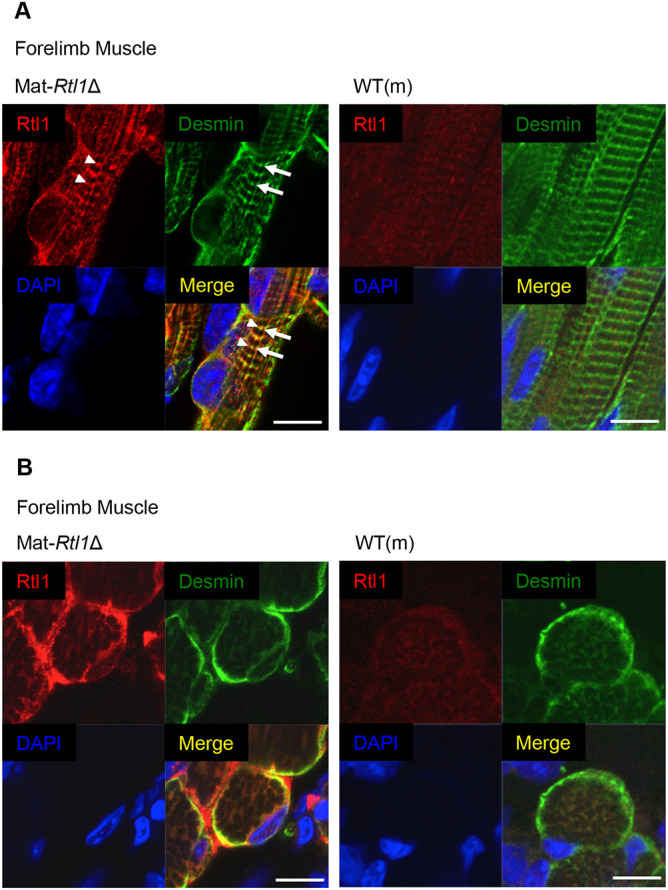
**Expression of *Rtl1* in the neonatal muscle.** (A,B) Immunofluorescence staining of RTL1 protein in the neonatal forelimb muscles from Mat*-Rtl1*Δ and WT(m) mice. Long axis views (A) and cross-sectional views (B) of the muscle fibers. Co-immunostaining with RTL1 (red; arrowheads), desmin (green; arrows) and DAPI (blue), and their merged images. Scale bars: 20 μm. The neonates were not fixed before being embedded in the OCT compound.

Immunofluorescence experiments using both longitudinal and cross-sections of these skeletal muscles demonstrated the localization of the RTL1 protein in the muscle fibers and the sarcolemma as well as around the muscle fiber nuclei (Fig. 3A,B). We found that RTL1 protein is expressed in a typically striped pattern in the striated muscle, displaying a pattern strikingly similar to that of α-actinin, which is located at the sarcomeric Z-disc (Clark et al., 2002; Sjöblom et al., 2008), although the signals of RTL1 were not evidently merged with α-actinin under high magnification (Fig. S8A,B). However, RTL1 seemed to be partially merged with desmin, which is known as an intermediate filament as well as one of a sarcomeric cytoskeleton showing some of the links between membranes (sarcolemma and nuclear membrane) and sarcomeres at the Z-disc. This confirms the precise location of RTL in the adjoining area of the Z-disc (Goldfarb and Dalakas, 2009) (Fig. 3A). Importantly, in the cross-section, both RTL1 and desmin localized on the surface of myofibrils at the Z-disc in an alternated manner but were not merged, demonstrating that these two proteins are located next to each other (Fig. 3B and Fig. S8C). It has been reported that desmin (*Des*) is the earliest marker in muscle development, detected around E9.0 (Fürst et al., 1989; Milner et al., 1996). However, *Des* KO mice are normal at birth but exhibit severe skeletal and cardiac muscle defects around 2 weeks after birth, leading to partial lethality (Milner et al., 1996; Li et al., 1997). This corresponds with the time point when *Rtl1* expression ceases in the skeletal muscles (Fig. 1). Thus, it is likely that RTL1 plays a specific role in the fetal/neonatal muscle fibers, such as stabilizing the muscle contractile apparatus and/or regulating muscle constriction, instead of (or together with) desmin. This seems consistent with the severe shrinkage of the muscle fibers observed in the Mat-*Rtl1*Δ mice after fixation (Fig. 2D and Fig. S7E).

### Rtl1 regulates satellite cell proliferation and determines the integrity of their differentiated myotubes

**Fig. 4.**
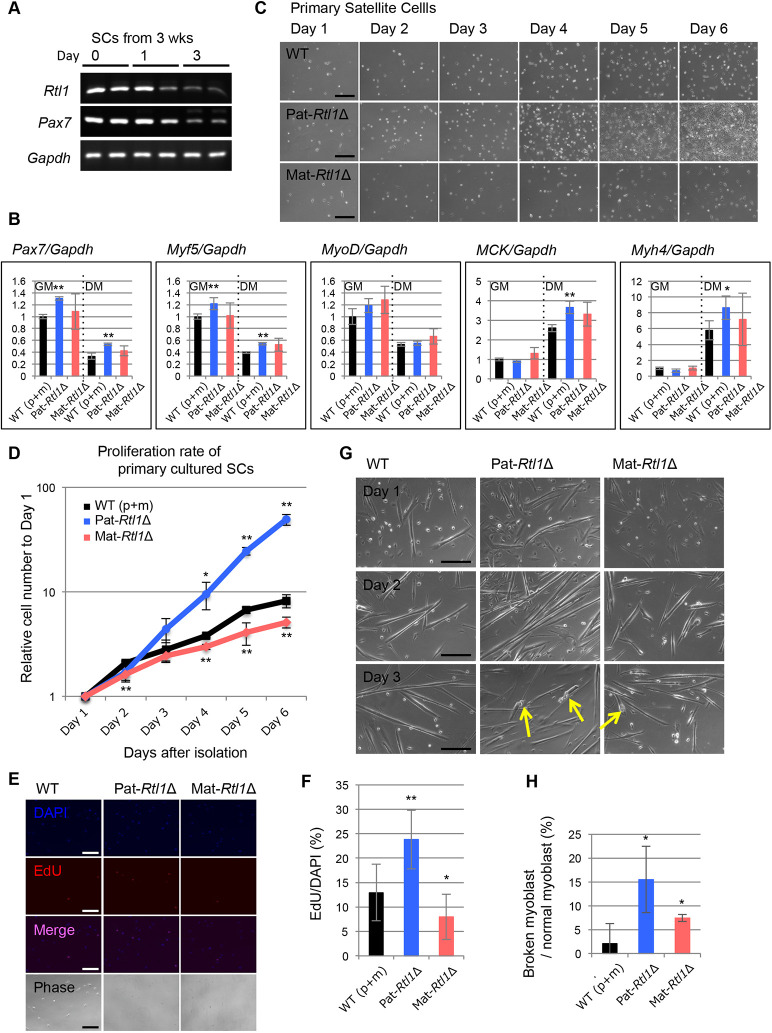
***Rtl1* expression during differentiation of SCs.** (A) Expression of *Rtl1* in primary cultured SCs. SCs during proliferation (day 0) and after the induction of differentiation (1 and 3 days) are shown. *Gapdh* was used as the control. Results after 35 cycles of amplification were shown. (B) Expression of several muscle marker genes in proliferating and differentiating SCs. SCs during proliferation (left; GM, growth medium) and 3 days after inducing differentiation (right; DM, differentiation medium) of wild type (black, *n*=6), Pat*-Rtl1*Δ (blue, *n*=3) and Mat-*Rtl*Δ (red, *n*=3). *Gapdh* was used as the control. *P<0.05, **P<0.01 (two-tailed Student's *t*-test). Data are mean±s.d. (C) Phase-contrast microscopic images of wild-type, Pat*-Rtl1*Δ and Mat*-Rtl1*Δ SC proliferation from day 0 to day 6. Scale bars: 200 μm. (D) *In vitro* cell proliferation assay of wild-type (black, *n*=6), Pat-*Rtl1*Δ (blue, *n*=3) and Mat-*Rtl1*Δ (red, *n*=3) SCs. *P<0.05, **P<0.01 (two-tailed Student's *t*-test). Data are mean±s.d. (E) EdU incorporation in primary SCs. Immunofluorescence staining of DAPI (blue) and EdU (red), and their merged and phase-contrast microscopic images. Scale bars: 200 μm. (F) Comparison of the growth rates of wild-type (black, *n*=12), Pat*-Rtl1*Δ (blue, *n*=6) and Mat*-Rtl1*Δ (red, *n*=6) SCs. *P<0.05, **P<0.01 (two-tailed Student's *t*-test. Data are mean±s.d. (G) Phase-contrast microscopic images of SCs inducing differentiation from day 1 to day 3. The arrows indicate detached and rounded myoblasts. Scale bars: 200 μm. (H) Comparison of the broken myoblast of the wild-type (black, *n*=10), Pat*-Rtl1*Δ (blue, *n*=5) and Mat*-Rtl1*Δ (red, *n*=5) SCs. *P<0.05 (two-tailed Student's *t*-test). Data are mean±s.d.

Consistent with the *in vivo* data, *Rtl1* expression was observed in undifferentiated proliferating muscle satellite cells (SCs), which are stem cells for skeletal muscle regeneration, obtained from mice at 3 weeks of age, as well as in SC-differentiated myoblasts (Fig. 4A) where levels of the muscle stem cell marker *Pax7* and the early muscle differentiation markers *Myf5* and *Myod1* were typically decreased, whereas the levels of the late muscle differentiation markers *MCK* (*Ckm*) and *Myh4* were increased (Relaix et al., 2005; Wang and Rudnicki, 2011; Buckingham and Rigby, 2014) (Fig. 4B). *Pax7*, *Myf5*, *MCK* and *Myh4* levels significantly increased in Pat-*Rtl1*Δ compared with wild type.

Importantly, *Rtl1* affects SC proliferation and the structural strength of the SC-differentiated myoblasts: SCs from the Pat-*Rtl1*Δ mice, which did not exhibit a substantial *Rtl1* expression, proliferated at a significantly higher rate (1.5-fold) than the control cells (Fig. 4C,D), whereas the SCs from the Mat-*Rtl1*Δ mice proliferated at a significantly lower rate (×0.8) (Fig. 4C,D). This result demonstrates that *Rtl1* has a repressive role in satellite cell proliferation, and its expression is required for normal growth. This finding was confirmed by EdU incorporation, which was significantly higher (×1.6) and lower (×0.7) than that in the controls (*P*=0.000857 and *P*=0.0369, two-tailed Student's *t*-test, respectively, Fig. 4E,F). We then counted the number of Pax7^+^ satellite cells in the muscles in the wild type, Pat-*Rtl1*Δ and Mat-*Rtl1*Δ mice, but there was no significant difference in their number (Fig. S9). Furthermore, the myoblasts that differentiated from the Pat-*Rtl1*Δ and Mat-*Rtl1*Δ SCs evidently displayed a weak or low structural strength of myoblast cells, because some of the myoblasts were detached from the culture dishes and exhibited a rounded shape (Fig. 4G, arrows, H). This finding may also explain why both the Pat-*Rtl1*Δ and Mat-*Rtl1*Δ mice had an abnormal neonatal muscle structure.

### Pat-*Rtl1*Δ and Mat-*Rtl1*Δ mice are good models for Temple and Kagami-Ogata syndromes

We confirmed that the expression of other imprinted genes in the same region was almost normal in these muscles (Figs S1 and S10). All of these results provide strong evidence that the loss and overproduction of *Rtl1* are responsible for the muscle abnormalities observed in the Pat-*Rtl1*Δ and Mat-*Rtl1*Δ mice, respectively, as well as the muscle symptoms observed in Temple and Kagami-Ogata syndromes (Kagami et al., 2005, 2008, 2015; Ogata and Kagami, 2016; Kotzot et al., 2004; Ioannides et al., 2014; Georgiades et al., 2000; Sekita et al., 2008; Kitazawa et al., 2017). Overall, there is a robust correlation between the muscle phenotypes observed in individuals with Temple and Kagami-Ogata syndromes and those in the Pat-*Rtl1*Δ and Mat-*Rtl1*Δ model mice (Kagami et al., 2005, 2008, 2015; Ogata and Kagami, 2016; Kotzot et al., 2004; Ioannides et al., 2014; Georgiades et al., 2000; Sekita et al., 2008; Kitazawa et al., 2017) (Table 1 and Tables S1-S3). The Pat-*Rtl1*Δ mice and mUPD12 mice exhibit late fetal/neonatal lethality, whereas individuals with Temple syndrome show no lethality; however, this is an apparent genetic background effect in the mice (Georgiades et al., 2000; Sekita et al., 2008; Kitazawa et al., 2017). The individuals with Temple syndrome frequently exhibit muscular hypotonia and feeding difficulty/poor sucking function in early childhood, consistent with the muscle abnormalities in the Pat-*Rtl1*Δ mice. Although there were no data reported, we speculate that abnormalities similar to those in Pat-*Rtl1*Δ mice may be observed in the intercostal muscle, diaphragm and abdominal muscle of human UPD(14)mat and mUPD12 mice. The muscle abnormalities in the Mat-*Rtl1*Δ mice are nearly consistent with those in the individuals with Kagami-Ogata syndrome, such as frequent abdominal wall hernia and diastasis recti. Although a bell-shaped narrow thorax is a typical symptom of Kagami-Ogata syndrome, only a mild rib bone phenotype was observed, even in the pUPD12 mice (Georgiades et al., 2000). We speculate that severe damage to the intercostal muscle might lead to the deformation of the rib bone in humans, because muscle contractions could control skeletal morphogenesis during development (Shwartz et al., 2012, 2013). It seems reasonable that the abnormalities observed in the *Rtl1* Pat- and Mat-*Rtl1*Δ mice would also be detected in the diaphragm, intercostal and abdominal muscles of human UPD(14)mat and UPD(14)pat patients.

## DISCUSSION

The *DLK1-DIO3* imprinted region is of considerable interest in terms of muscle development, but it is also very complicated. Many reports have indicated a relationship between imprinted genes in this region and muscle hypertrophy (the *callipyge* phenotype in sheep) and/or muscle development, especially paternally expressed *DLK1*, maternally expressed *MIRG*, a non-coding mRNA encoding the *miR-379/miR-410* cluster containing 39 miRNAs, and paternally expressed *RTL1* and *RTL1as* encoding the *miR-127/miR-136* cluster containing the seven miRNAs in this study. The *miR-379/miR-544* cluster (the anterior half of the *miR-379/miR-410* cluster) is highly expressed in neonatal muscle. Mice with a deletion of the *miR-379/miR-544* region upon maternal transmission exhibited significantly larger fast-switch muscles and MyHC type II-B fibers at P10 (Gao et al., 2015). In particular, *miR-329* in the *miR-379/miR-544* cluster has a regulatory role in *Dlk1* expression (Gao et al., 2015). In this case, the expression of *Rtl1* mRNA and RTL1 protein in the muscle tissues was comparable with wild type, implying no involvement of RTL1 in the hypertrophic phenotypes in these KO mice. Wüst et al. have also reported *miR-1/miR133a*-mediated inhibition of the *Dlk1-Dio3* mega gene cluster. They demonstrated that the deletion of *miR-1/miR133a* clusters caused abnormally high expression of the *miR-379/miR-544* cluster (encoded by *Rian* and *Mirg* in this report) as well as the *Meg3* (*Gtl2*) miRNA cluster during muscle differentiation mediated via *Mef2a* upregulation. As several miRNAs in the *miR-379/miR-544* cluster have the ability to target multiple mitochondria genes, the *miR-1/miR133a* deletion results in an abnormal metabolic maturation of the skeletal muscles (Wüst et al., 2018). However, by comprehensive miRNA analysis during skeletal muscle lineage progression, Castel et al. proposed that the *miR-127/miR-136* cluster in *Rtl1as* may be more important for the differentiation, proliferation, commitment to myogenesis and self-renewal of SCs, because the SCs isolated from mice with a deletion of the entire *miR-379/miR-410* cluster exhibit no differences in these abilities (Castel et al., 2018). We previously analyzed several patients with Kagami-Ogata syndrome-like phenotype without upd(14)pat and demonstrated that epimutations (hyper-DNA methylation) of the imprinting control region, the inter-genetic differentially methylated region (IG-DMR) of the *DLK1-DIO3* imprinted region and the maternal microdeletion of the IG-DMR also cause Kagami-Ogata syndrome-like phenotypes (Kagami et al., 2008). The IG-DMR deletions sometimes extend into neighboring imprinted gene region. We also demonstrated that the severity of Kagami-Ogata syndrome exhibits good correlation with the degree of *RTL1* overexpression but does not have any apparent correlation with the expression levels of *DLK1*, *MIRG* or other miRNA clusters (Kagami et al., 2008).

This study provides strong support that the loss and overproduction of human *RTL1* are the major cause of the muscle symptoms observed in Temple syndrome and Kagami-Ogata syndrome, respectively. We cannot rule out the possibility that some of the miRNA(s) in the *miR-127/miR-136* cluster in *Rtl1as* have different target gene(s) other than *RTL1* and are involved in muscle development. For example, it has been reported that *miR-431* in the *miR-127/miR-136* cluster promotes myogenic differentiation by targeting *Pax7* (Wu et al., 2014). The authors showed that *miR-431* regulates the *Pax7* levels during muscle development and regeneration using two *miR-431* TG strains of mice (4- and 20-fold overexpression of *miR-431*, respectively), but there was no difference in *Rtl1* expression in the skeletal muscle of *miR-431* transgenic and wild-type mice. They concluded that *miR-431* regulates myogenic differentiation independently of *Rtl1*. However, in our models, the *Pax7* expression level was not affected in the Mat*-Rtl1*Δ SCs (without *miR-431* and other five miRNAs), whereas it was upregulated in the Pat*-Rtl1*Δ SCs (Fig. 4B), indicating that the phenotypes we observed in this study are independent of the *miR-431-Pax7* pathway.

In our mouse models, the damage to the muscles essential for respiratory function, such as the intercostal, diaphragm and abdominal muscles, was more severe than in the limb muscles in neonates. It is highly likely that these defects were the major cause of neonatal lethality in knockout mice models, thus indicating that the loss of *RTL1* is a major cause of muscular hypotonia and feeding difficulty/poor sucking function seen in Temple syndrome, and that the overproduction of *RTL1* induced by loss of *RTL1as* is a major cause of the respiratory problems and diastasis recti damage in Kagami-Ogata syndrome.

As mentioned above, individuals with Temple syndrome have muscle abnormalities consistent with those in the Pat-*Rtl1*Δ mice (Kotzot, 2004; Ioannides et al., 2014; Kagami et al., 2017). The phenotypes of the Pat-*Rtl1*Δ mice are more severe than those in individuals with Temple syndrome because the Pat-*Rtl1*Δ mice exhibit late fetal/neonatal lethality (Sekita et al., 2008; Kitazawa et al., 2017), whereas the Temple syndrome patients show no lethality (Kagami et al., 2008). However, this is an apparent genetic background effect in the mice because the severity gradually increases with the ratio of the B6 genotype, whereas F1 mice crossed with B6 and other strains do not exhibit any neonatal lethality, but instead exhibit both neo- and postnatal growth retardation patterns similar to those in individuals with Temple syndrome (Sekita et al., 2008; Kitazawa et al., 2017). Individuals with Temple syndrome lack any *DLK1* and *RTL1* expression. It is known that *Dlk1* KO mice also exhibit growth retardation and neonatal lethal phenotypes to some extent (Moon et al., 2002), and overexpression of *Dlk1* causes muscular hypertrophy (Davis et al., 2004). Loss of paternally inherited *DLK1* is reported to frequently cause precocious puberty in females with Temple syndrome (Dauber et al., 2017). Therefore, it is clear that combined and differential involvement of *DLK1* and *RTL1* should be considered when investigating the symptoms of Temple syndrome.

As we mentioned in the Results, the muscle abnormalities in the Mat-*Rtl1*Δ mice are also consistent with the fact that abdominal wall hernia and diastasis recti frequently occur in individuals with Kagami-Ogata syndrome. Although a bell-shaped narrow thorax is a typical symptom of Kagami-Ogata syndrome (Kagami et al., 2005, 2008, 2015), only a mild rib bone phenotype was observed, even in the pUPD12 mice (Georgiades et al., 2000). We have previously demonstrated that the severity exhibits good correlation with the degree of *RTL1* overexpression (Kagami et al., 2008). We speculate that severe damage to the intercostal muscle might lead to the deformation of the rib bone in humans, because muscle contractions control skeletal morphogenesis during development (Shwartz et al., 2012, 2013). Thus, the intercostal, diaphragm and abdominal muscle defects, along with the resulting bell-shaped narrow thorax, presumably reflect the evident neonatal respiratory problems. Recently, Loo et al. reported that *RTL1* is involved in muscle regeneration under the control of the linker of nucleoskeleton and cytoskeleton (LINC) complex component SUN1 (Loo et al., 2019). We also observed the re-expression of *RTL1* in regenerating muscles (Fig. S11), indicating an involvement of RTL1 in the basic eutherian muscle generation program. We also observed the re-expression of *Rtl1* in regenerating muscles, suggesting a possible role for *Rtl1* in muscle regeneration as well as in muscle generation in eutherians, although the mechanism of RTL1 in these processes is unknown and future studies should be directed to mechanistic biochemical and genetic investigations.

Our study clearly demonstrates that RTL1 is of crucial physiological significance because it plays a major role in the maturation and maintenance of fetal muscle fibers (Figs 2 and 4); therefore, its loss and overproduction affect the muscle phenotypes of Temple and Kagami-Ogata syndromes, respectively (Table 1 and Tables S1-S3). How, then, does RTL1 work in the fetal/neonatal muscle fibers? An immunostaining experiment revealed that RTL1 is closely located to desmin at the level of the Z-disc (Fig. 3). Desmin is part of the sarcomeric cytoskeleton and is involved in linking membranes and sarcomeres at the Z-disc, acting as a sub-sarcolemmal protein that is part of the costamere – a structural-functional component of the striated muscle cells at the periphery of the Z-disc (Goldfarb and Dalakas, 2009). This muscle-specific complex plays an important role in connecting the force-generating sarcomeres with the sarcolemma, which helps to couple the sarcomere to the extracellular matrix (ECM). Thus, it is likely that the RTL1 protein plays a specific role in the function of fetal/neonatal muscle fibers, such as stabilizing the muscle contractile apparatus and/or regulating muscle constriction with desmin. Therefore, its loss and overproduction affect the strength of muscle fibers, as shown in Figs 2G and 4G. We also speculate that, at normal expression levels, the RTL1 protein also plays a role as a suppressor of desmin to prevent fast and vigorous muscle movement in the fetal/neonatal stages by interacting or interfering with desmin. This speculation is supported by the fact that desmin KO mice exhibit no gross abnormality in the fetal and neonatal periods; rather, they exhibit severe muscle defects around 2 weeks after birth (Milner et al., 1996; Li et al., 1997). Thus, it is possible that desmin starts functioning at the Z-disc with the disappearance of the RTL1 protein at the Z-disc. This suggests that no Rtl1 expression is observed in the heart throughout development (Fig. 1A,B), because the cardiac muscles must continuously function after the formation of the heart at E8.5. This view appears to be consistent with the observation that human babies and mouse pups exhibit slow and weak muscle movement immediately before and after birth. This type of inhibitory function of RTL1 in the fetal and neonatal muscles seems advantageous for both mothers, fetuses and neonates to ensure a safe pregnancy and to aid child-rearing in the current eutherian reproductive system. Our study also demonstrates that the eutherian skeletal muscle is unique because of the recruitment of RTL1 in fetal and neonatal muscle development, implying that it was one of the adaptations to the mammalian viviparaous reproduction system.

Despite its evolutionary origin as an LTR retrotransposon, *RTL1* also plays an essential role in the maintenance of placental fetal capillaries in adapting to the long gestation period in the current eutherian reproduction system as a eutherian-specific gene (Sekita et al., 2008; Kagami et al., 2008; Kitazawa et al., 2017; Charlier et al., 2001; Edwards et al., 2008). Thus, this work provides additional evidence that LTR retrotransposons have exerted a profound impact on a variety of eutherian-specific traits, including the skeletal muscles as well as the placenta and the brain (Sekita et al., 2008; Kitazawa et al., 2017; Edwards et al., 2008; Ono et al., 2006; Naruse et al., 2014; Irie et al., 2016) and that ‘gene acquisition from exogenous DNAs’ is a crucial driving force in therian/eutherian evolution (Irie et al., 2015; Gould and Vrba, 1982; Brosius and Gould, 1992; Kaneko-Ishino and Ishino, 2012, 2015).

## MATERIALS AND METHODS

### Mice

All animals and experimental procedures were approved by the Animal Ethics Committees of Tokyo Medical and Dental University. The *Rtl1* KO mice were generated by using ES cells (CCE) of 129/SvEv mouse origin, as previously described (Sekita et al., 2008). The *Rtl1* KO lines (Mat-*Rtl1*Δ and Pat-*Rtl1*Δ) were maintained by continuous crossing with male and female C57BL/6J mice (WT), respectively, and mice in the F8 (*Rtl1* KO: Pat-*Rtl1*Δ) and F9 (*antiRtl1as* KO: Mat-*Rtl1*Δ) generations were used in this study.

### Analysis of the expression of *Rtl1*

The genomic DNA and total RNA samples were prepared from fetuses, neonates and 1- to 2-week-old mice by using TRIzol Regent (Life Technologies). cDNA was synthesized from 1-5 μg of total RNA using SuperScript III Reverse Transcriptase (Invitrogen) with the following oligo-dT+ adaptor primer: 5′-CTGATCTAGAGGTACCGGATCCGACTCGAGTCGACATCGTTTTTTTTTTTTTTTTT-3′. For the RT-PCR analysis of *Rtl1*, 10 ng of cDNA in a 25 μl reaction mixture containing 1×KOD FX buffer (KFX-101, TOYOBO), 200 μM of each dNTP, primers and 0.5 units of KOD FX were subjected to 30 cycles at 98°C for 15 s, 69°C for 30 s and 74°C for 30 s using a C1000 Touch thermal cycler (BioRad). The following primer sequences were used: *Rtl1*, 5′-TCCAAGGAGCATTCGACGTACCAGTGTGACTTACC-3′; an adaptor primer for both genes, 5′-AGAGGTACCGGATCCGACTCGAGTCGACATCG-3′; and *Gapdh*, 5′-CACTCTTCCACCTTCGATGC-3′ and 5′-CTCTTGCTCAGTGTCCTTGC-3′.

### Quantitative PCR assay

The quantitative real-time PCR was performed using 5 ng of cDNA in a THUNDERBIRD SYBR qPCR Mix (QPS-201, TOYOBO). The cycle conditions were 95°C for 1 min, followed by 40 cycles of 95°C for 10 s, 60°C for 20 s and 72°C for 10 s using the LightCycler 480 apparatus (Roche). The gene expression levels were normalized to *Gapdh*. The following primer sequences were used for this study: *Rtl1*, 5′-GAGTACTGTGCCAAGGAGCC-3′ and Adaptor primer; *Dlk1/Peg9*, 5′-TTACCGGGGTTCCTTAGAGC-3′ and 5′-TGCATTAATAGGGAGGAAGGG-3′; *Meg3/Gtl2*, 5′-TTGCACATTTCCTGTGGGAC-3′ and 5′-AAGCACCATGAGCCACTAGG-3′; *Meg8/Rian*, 5′-TCGAGACACAAGAGGACTGC-3′ and 5′-ATTGGAAGTCTGAGCCATGG-3′; *Meg9/Mirg*, 5′-TTGACTCCAGAAGATGCTCC-3′ and 5′-CCTCAGGTTCCTAAGCAAGG-3′; and Dio3, 5′-TCGAGATAGGGAAAGGGTGG-3′ and 5′-GAACCTCGCAGATTGATTCC-3′.

### Western blotting

The protein samples were extracted from E16.5 fetuses using 8 M urea, 2% CHAPS and cOmplete EDTA-free protease inhibitor cocktail (Roche). We loaded 3 μg of total protein into the well of the 7.5% SDS-PAGE gel and ran the gel for 35 min at 200 mV. We transferred the protein from the gel to the PVDF membrane and the PVDF membrane was blocked with 5% skim milk in TBS-T at 4°C overnight. We incubated the membrane with rabbit anti-Rtl1 antibody (1:200; Sekita et al., 2008) in TBS-T as the primary antibody at room temperature for 2 h and with anti-rabbit IgG, with an HRP-linked antibody as the secondary antibody (1:5000; Cell Signaling Technology, #7074) in TBS-T at room temperature for 1 h. Western blotting was performed using ECL Select Western Blotting Detection Reagent (GE Healthcare).

### Histological analysis (paraffin sections)

Mouse fetuses and neonates were fixed by using Super Fix (KURABO), soaked in 5% formic acid in 70% ethanol at 4°C overnight for 2 nights; dehydrated in 70% and 90% ethanol for 2 h each, in 100% ethanol for 2 h three times, and in xylene for 2 h four times; and finally embedded in paraffin wax. The paraffin blocks were sectioned at 5 μm with a microtome and mounted on Superfrost Micro Slides (Matsunami Glass). The sections were deparaffinized three times in xylene for 20 min, three times in 100% ethanol for 5 min, and in 90% and 70% ethanol for 5 min each; the sections were then stained with Hematoxylin for 10 min, and washed with tap water for 2 min. After being stained with Eosin for 1 min, the sections were immersed once in 70% and 80% ethanol for a few seconds each and three times in 100% ethanol for 3 min, then dehydrated three times in xylene for 3 min and mounted with Malinol mounting medium (MUTO).

Another section was stained with resorcin-fuchsin solution for 1 h, immersed in 100% ethanol for a few seconds and washed with tap water for 10 min. After being stained with Weigert iron Hematoxylin solution for 5 min, the sections were washed with tap water for 7 min. The sections were then stained with van Gieson solution for 10 min and immersed once each in 70% and 80% ethanol for a few seconds and three times in 100% ethanol for 3 min, then dehydrated three times in xylene for 2 min and mounted with Malinol mounting medium.

### Immunostaining (paraffin sections)

For antigen retrieval, the sections were boiled in Immunosaver (1:200; Nissin EM) at 98°C for 40 min and then immersed (dehydrated) in ice-cold methanol at −30°C overnight. After being air dried, the sections were blocked with 10% goat serum, 1% bovine serum albumin (BSA: Sigma Aldrich) and 0.1% Triton-X 100 (WAKO) in PBS at room temperature for 1 h.

For the immunohistochemistry analysis, anti-Rtl1 antibody (1:200) was used as the primary antibody and was prepared in 1% BSA and 0.1% Triton-X 100 in PBS at 4°C overnight (for more than 20 h). This primary reaction was developed with a biotinylated goat anti-rabbit IgG secondary antibody (1:200; Vector Laboratories) for 2.5 h, then incubated with alkaline phosphatase (AP) complex (1:200; Vector Laboratories) for 1 h. The histochemical detection of the alkaline phosphatase activity was performed with BCIP/NBT (Vector Laboratories) in 100 mM Tris-HCl at pH 9.8 and mounted with VectaMount AQ Mounting Medium (Vector Laboratories). The images were captured using BIOREVO (Keyence).

### Histological analysis (cryosections)

The mouse neonates were corrected and embedded in OCT compound (Sakura Finetek). The OCT blocks were sectioned using a cryostat (MICROME), at 14 μm and mounted on Superfrost Micro Slides. The cryosections were fixed in 4% paraformaldehyde (PFA: Nacalai tesque) for 10 min at room temperature and washed three times with PBS for 5 min, then stained with Hematoxylin for 2 min and washed with tap water for 2 min. After being stained with Eosin for 1 min, the slides were immersed once in 70% and 80% ethanol for a few seconds each and three times in 100% ethanol for 2 min, then dehydrated three times in xylene for 3 min and mounted with Malinol mounting medium.

### Immunostaining (cryosections)

The cryosections were fixed in 4% PFA for 10 min at room temperature and washed three times with PBS for 5 min. For the antigen retrieval, the sections were boiled in 0.01 M citric acid solution (pH 6.0) at 80°C for 10 min and then immersed (dehydrated) in ice-cold methanol at −30°C for 6 min. After being air dried, the sections were blocked with 10% goat serum, 5% BSA and 0.5% TritonX-100 in PBS at room temperature for 1 h, and then incubated with the primary antibody, which was prepared in 5% BSA and 0.5% TritonX-100 in PBS at 4°C overnight (∼16 h).

For the immunofluorescence staining, anti-Rtl1 antibody (1:200, Sekita et al., 2008), anti-desmin antibody (Roche, DE-R-11, non-diluted), anti-Laminin antibody (1:200; Sigma Aldrich, L9393), anti-α-actinin (sarcomeric) antibody (1:100; Sigma Aldrich, A7811) and Pax7 (1;100; Santa Cruz, sc-81648) were used as the primary antibodies. Alexa Fluor 488-conjugated anti-mouse IgG (1:1000; ThermoFisher Scientific, A11008) and 555-conjugated anti-rabbit IgG (1:1000; ThermoFisher Scientific, A28180) or 488-conjugated anti-rabbit IgG (1:1000; ThermoFisher Scientific, A11034) were used as the secondary antibodies and stained with DAPI (1:1000; Wako, 340-07971) for 1 h. The slides were mounted in VectaShield (Vector Laboratories). The images were captured with an LSM710 confocal imaging system (Zeiss).

### Satellite cells isolation from 3-week-old mice and culture

Satellite cells were isolated from the anterior tibialis anterior (TA) and gastrocnemius muscles of 3-week-old male mice. The muscle fragments were incubated in medium A [GlutaMax-DMEM (Gibco), containing 1% penicillin-streptomycin (Sigma Aldrich)] at 37°C for 30 min, then minced well. The minced muscles were digested in medium B [medium A containing 0.14% protease (Sigma Aldrich)] at 37°C for 10 min. After centrifugation at 400 rpm (30 ***g***) for 30 s, the supernatant was discarded. The pellet was resuspended in medium B and incubated at 37°C for 10 min. After centrifugation at 400 rpm (30 ***g***) for 30 s, the supernatant was collected. This procedure was repeated two additional times. The collected supernatant was filtered through a 100 μm cell strainer (Falcon), mixed with medium C [medium A containing 10% fetal bovine serum (FBS; Gibco)] and centrifuged at 2000 rpm (400 ***g***) for 5 min. The pellet was resuspended in 10 ml of medium C and centrifuged one more time. The pellet was then resuspended in growth medium [GM; DMEM-GlutaMax with 20% FBS, 1% penicillin-streptomycin, 1% chicken embryonic extract (CEE; US Biological) and 10 ng/ml basic fibroblast growth factor (bFGF; Falcon)] and seeded onto Matrigel Matrix (Falcon)-coated dishes. The cells were cultured at 37°C under 5% CO_2_ in a humidified chamber, and the medium was changed every 2 days.

### Analysis of the expression of muscle markers in the satellite cells

Total cellular RNA were isolated using a Qiagen RNeasy kit according to the manufacturer's protocol. The cDNA was synthesized from 250 ng of total RNA using SuperScript III Reverse Transcriptase with the oligo-dT+ Adaptor primer. For the RT-PCR, 10 ng of cDNA in a 25 μl reaction mixture containing 1× Ex Taq buffer, 200 μM of each dNTP, primers and 0.5 units of Ex Taq Hot Start were subjected to 30 cycles at 96°C for 15 s, 60°C for 20 s and 72°C for 30 s in a C1000 thermal cycler (BioRad). The following primer sequences were used: *Pax7*, 5′-AGGCCTTCGAGAGGACCCAC-3′ and 5′-CTGAACCAGACCTGGACGCG-3′; *Myf5*, 5′-TGAAGGATGGACATGACGGACG-3′ and 5′-TTGTGTGCTCCGAAGGCTGCTA-3′; *Myod1*, 5′-GGCTACGACACCGCCTACTA-3′ and 5′-GAGATGCGCTCCACTATGCT-3′; myogenin, 5′-AGTGAATGCAACTCCCACAG-3′ and 5′-ACGATGGACGTAAGGGAGTG-3′; *MCK*, 5′-CTGACCCCTGACCTCTACAAT-3′ and 5′-CATGGCGGTCCTGGATGAT-3′; *Myh4*, 5′-GCTTGAAAACGAGGTGGAAA-3′ and 5′-CCTCCTCAGCCTGTCTCTTG-3′; *Gapdh*, 5′-CACTCTTCCACCTTCGATGC-3′ and 5′-CTCTTGCTCAGTGTCCTTGC-3′.

### Muscle regeneration

To induce muscle regeneration, 100 μl of 10 mM cardiotoxin (CTX; C9759, Sigma-Aldrich) were injected intramuscularly into the tibialis anterior (TA) muscle of anesthetized 10-week-old male mice using a 29 G syringe. Regenerating muscles were isolated 2, 4 and 7 days after CTX injection.

## Supplementary Material

Supplementary information

Reviewer comments
