# Supplementary material for: Deficiency and overexpression of Rtl1 in the mouse cause distinct muscle abnormalities related to Temple and Kagami-Ogata syndromes
Source: Development. 2020 Sep 3;147(21):dev185918. doi: 10.1242/dev.185918 (PMC7490516; doi:10.1242/dev.185918)
Supplement: Supplementary information [file develop-147-185918-s1.pdf]

Table S1. Abdomenal abnormalities reported in human upd(14)pat patients.

This case	Abdomen
Mattes et al. 2007	Diastasis recti, hepatomegaly
Curtis et al. 2006	Protruding abdomen
Kagami et al. 2005	Diastasis recti
Kagami et al. 2005	Omphalocele
Kagami et al. 2005	Diastasis recti
Stevenson et al. 2004	Diastasis recti, gastrostomy
Kurosawa et al. 2002	Diastasis recti, hypoplastic ilia
Kurosawa et al. 2002	Diastasis recti, hypoplastic ilia
Kurosawa et al. 2002	Diastasis recti, hypoplastic ilia
Towner et al. 2001, Coveler et al. 2002	Ventral hernias, omphalocele, undescended testes
McPherson et al. 2001, Chu et al. 2004	Diastasis recti, hypospadias, short ilia
Yano et al. 2001	Abdominal wall hernias
Cotter et al. 1997	Abdominal wall hernia, hepatosplenomegaly
Papenhausen et al. 1995	Abdominal wall hernia

・Diastasis recti	8
・Omphalocele	2
・Ventral hernia	1
・Abdominal wall hernia	3

Total number of patients = 19

The lower part of the column has a summary of the abdomenal abnormalities.

Development • Supplementary information

Table S2. Thorax abnormalities reported in human upd(14)pat patients.

This case	Thorax
Mattes et al. 2007	Small bell-shaped thorax, coat hanger shaped ribs, respiratory failure and need ventilation, elongated clavicles, lung hypoplasia, pulmonary hypertension, increased secretions
Curtis et al. 2006	Small bell-shaped thorax, coat hanger shaped ribs, respiratory failure and need ventilation
Kagami et al. 2005	Small bell-shaped thorax, coat hanger shaped ribs, respiratory failure and need ventilation
Kagami et al. 2005	Small bell-shaped thorax, coat hanger shaped ribs, respiratory failure and need ventilation, ASD
Kagami et al. 2005	Small bell-shaped thorax, coat hanger shaped ribs, respiratory failure and need ventilation, laryngomalacia
Stevenson et al. 2004	Coat hanger shaped ribs, respiratory failure, narrow scapular neck, inverted nipples, increased oral secretions, pulmonary artery stenosis
Offiah et al. 2003	Small bell-shaped thorax, coat hanger shaped ribs, cupped anterior ends of ribs, widely spaced nipples, absent glenoid fossae, abnormal costochondral junctions, VSD
McGowan et al. 2002	Pectus excavatum, respiratory failure, hypertrophic cardiomyopathy, curved ribs
Kurosawa et al. 2002	Narrow thorax, short wavy ribs, respiratory failure and ventilation
Kurosawa et al. 2002	Narrow thorax, short wavy ribs, respiratory failure and ventilation
Kurosawa et al. 2002	Narrow thorax, short wavy ribs, respiratory failure and ventilation
Towner et al. 2001, Coveler et al. 2002	small bell-shaped thorax, respiratory failure and ventilation, Pulmonary hypoplasia, pulmonary stenosis, ASD, VSD
McPherson et al. 2001, Chu et al. 2004	Bell-shaped thorax, curved ribs, respiratory failure
Cotter et al. 1997	Small and bell-shaped thorax, curved ribs, ventilation,elongated clavicles, restrictive lung disease, cardiomyopathy
Walter et al. 1996	Small and bell-shaped thorax, abnormal ribs, ventilation, prominent sternum, hypoplastic nipples, hypertrophic cardiomyopathy, ASD
Wang et al. 1991	Small and bell-shaped thorax, angulated ribs, restrictive lung disease

· Small bell-shaped thorax	11 (9)
· Narrow thorax	3 (3)

Total number of patients = 19
( ): respiratory failure

The lower part of column has a summary of thorax abnormalities. Eleven of the 19 patients exhibited a small, bell-shaped thorax including 9 cases of respiratory failure. Three of the 19 patients exhibited a narrow thorax, including 3 cases of respiratory failure.

Table S3. Musculoskeletal abnormalities reported in human upd(14)mat patients.

This case	Musculoskeletal
Gillessen-Kaesbach et al. 2018	Hypotonia, feeding problems, motor delay
Zhang et al. 2016	Hypotonia, feeding difficulties, scoliosis
Stalman et al. 2015	Hypotonia, motor developmental delay, hyperextensible joints
Hosoki et al. 2009	Hypotonia
Mitter et al. 2006	Hypotonia, feeding problems, motor delay, hyperextensible joints
Cox et al. 2004	Hypotonia, feeding problems
Manzoni et al. 2000	Motor delay
Martin et al. 1999	Hypotonia, motor and speech delays, joint laxity
Hordijk et al. 1999	Hypotonia, feeding problems, motor delay
Berends et al. 1999	Hypotonia, feeding problems, motor delay, scoliosis, joint laxity
Miyoshi et al. 1998	Hypotonia, feeding problems, motor delay, scoliosis
Splitt and Goodship. 1997	Motor deray, scoliosis
Tomkins et al. 1996	Hypotonia, feeding problems, motor delay
Coviello et al. 1996	Hypotonia, motor delay, scoliosis
Healey et al. 1994	Motor delay
Robinson et al. 1994	Hypotonia, motor retardation
Antonarakis et al. 1993	Hypotonia, feeding difficulties, motor delays, scoliosis, hyperextensible joints
Pentao et al. 1992	Diffuse hypotonia, motor retardation
Temple et al. 1991	Poor motor coordination, scoliosis

・Hypotonia	15
・Feeding problems	9
・Motor delay	16
・Scoliosis	7
・Hyperextensible joints	5

Total number of patients = 19

In the lower part of the column is a summary of the musculoskeletal abnormalities.

Development • Supplementary information

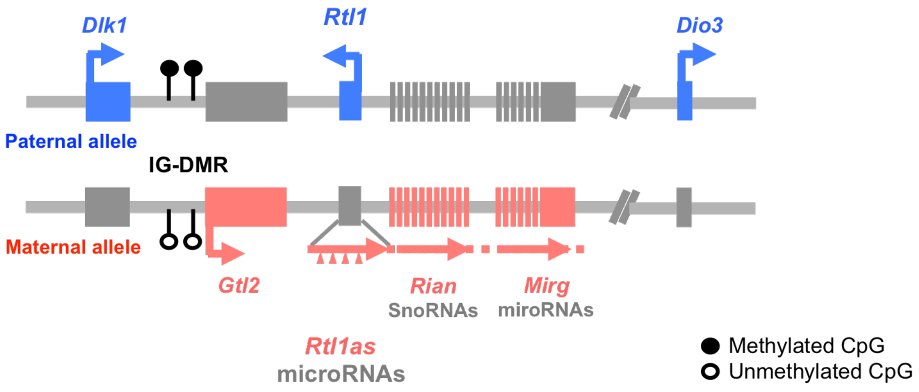

Fig. S1. Imprinted genes in the distal chromosome 12 in mice.

There are three coding *Pegs* (blue) and four non-coding *Megs* (red).

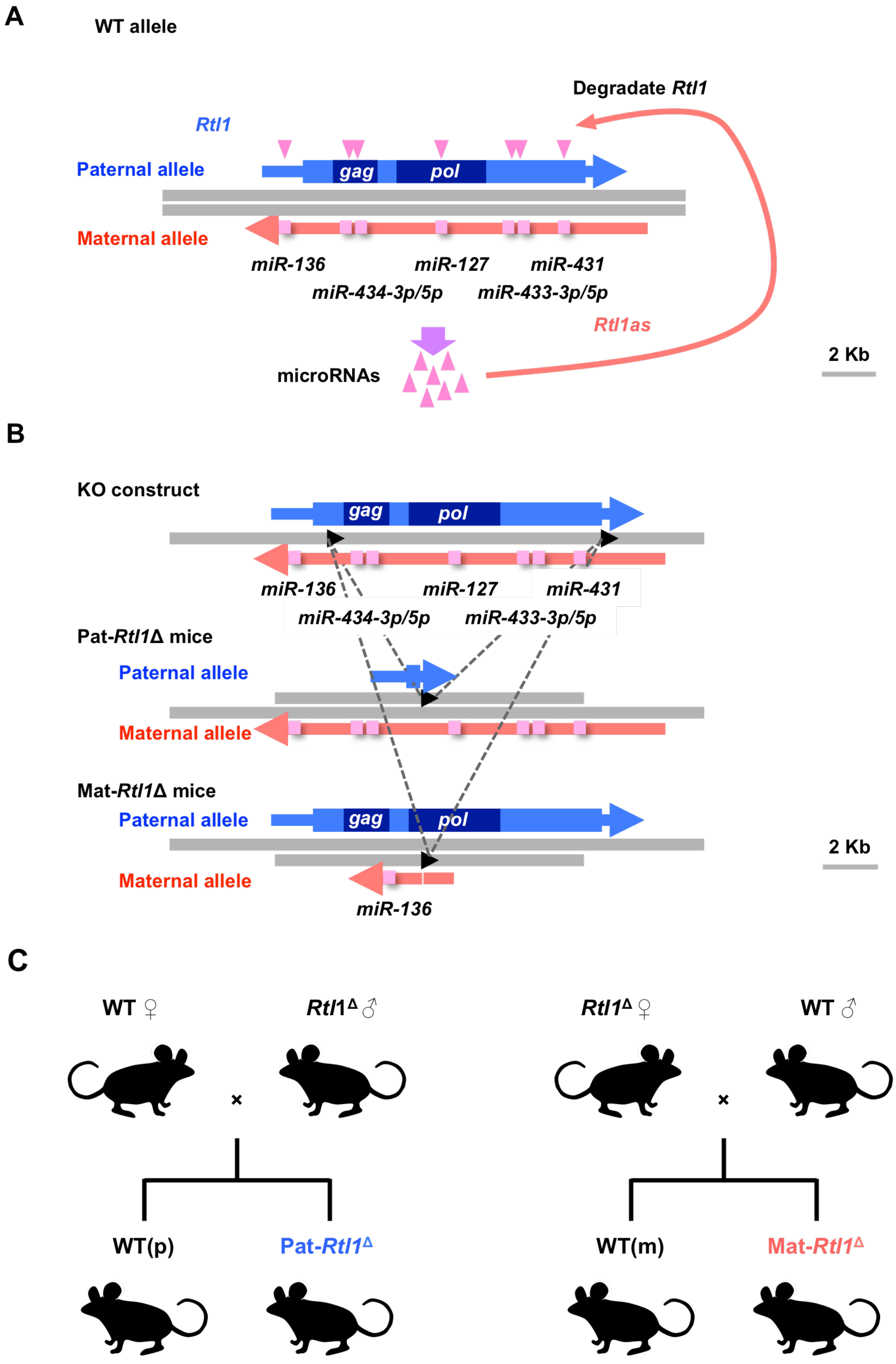
A
WT allele
Degradate Rtl1
Rtl1
Paternal allele
gag
pol
Maternal allele
miR-136
miR-127
miR-431
miR-434-3p/5p
miR-433-3p/5p
Rtl1as
2 Kb
microRNAs
B
KO construct
gag
pol
miR-136
miR-127
miR-431
miR-434-3p/5p
miR-433-3p/5p
Pat-Rtl1Δ mice
Paternal allele
Maternal allele
Mat-Rtl1Δ mice
Paternal allele
gag
pol
2 Kb
Maternal allele
miR-136
C
WT ♀
Rtl1Δ ♂
Rtl1Δ ♀
WT ♂
×
×
WT(p)
Pat-Rtl1Δ
WT(m)
Mat-Rtl1Δ

Fig. S2. Construction of *Rtl1* KO animals.

(A) The miRNAs in *AntiRtl1as* degrade *Rtl1* mRNA. **(B)** Construction of *Rtl1* KO animals. **(C)** A schematic representation of the breeding strategies used to generate Pat-*Rtl1*Δ and Mat-*Rtl1*Δ individuals.

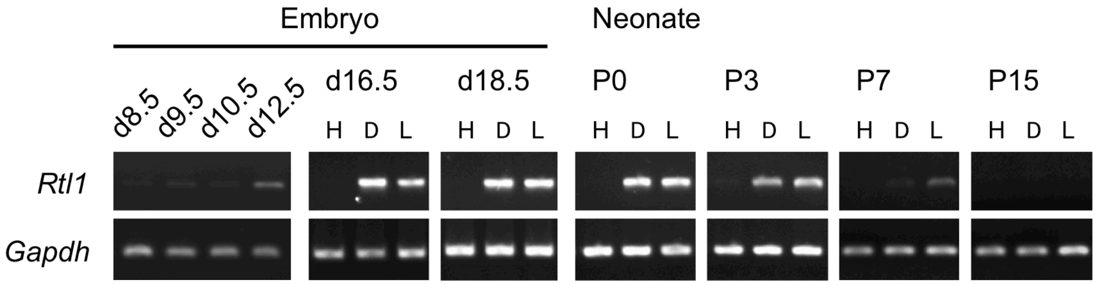

Fig. S3. *Rtl1* mRNA expression of in embryos, embryonal and neonatal muscles. RT-PCR of *Rtl1* in the whole embryo (d8.5-12.5), Heart (H), diaphragm (D), limb muscles (hindlimb: L) in d16.5 and d18.5 embryos and neonates (P0, P3, P7 and P15). *Rtl1* was expressed at least from d12.5 in embryos. *Gapdh* was used as the control. Results after 30 cycles of amplification were shown.

Development • Supplementary information

A

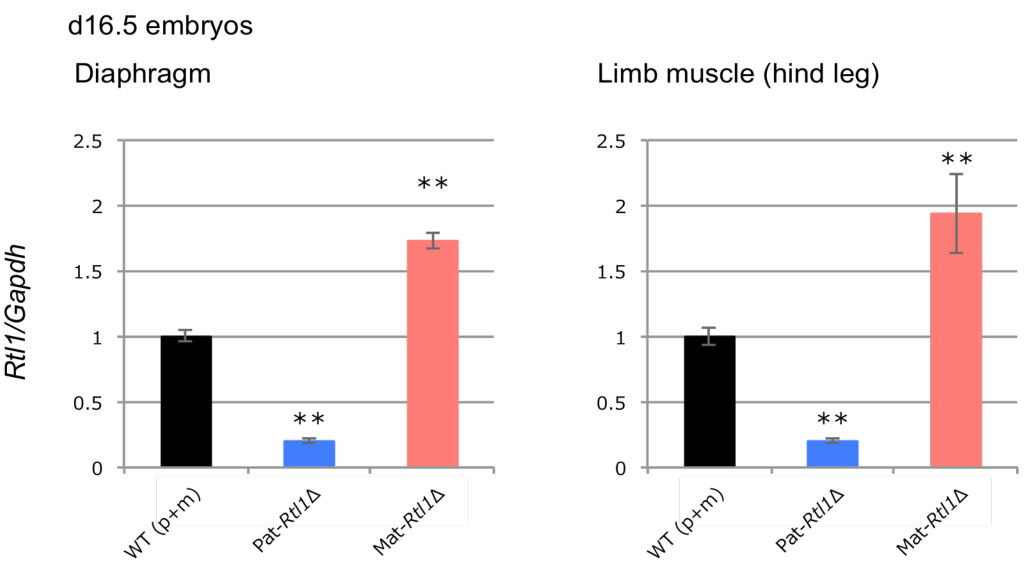

B

```
Mus musculus isolate MEA1001_3D_6F retrotransposon-like 1 (Rtl1) mRNA, complete cds
Sequence ID: EU434918.1  Length: 6375  Number of Matches: 1

Range 1: 6053 to 6375  GenBank  Graphics          ▼ Next Match  ▲ Previous Match

Score            Expect    Identities      Gaps        Strand
590 bits(319)    2e-164    322/323(99%)    1/323(0%)   Plus/Plus

Query  2     TGGACTGAT-CACCCTGAAGGTCCCTGAGACTCAGAGGACCCAAGAAAGGGTGGTAGCAA  60
             ||||||||| ||||||||||||||||||||||||||||||||||||||||||||||||||
Sbjct  6053  TGGACTGATCCACCCTGAAGGTCCCTGAGACTCAGAGGACCCAAGAAAGGGTGGTAGCAA  6112

Query  61    CCAAGTGGGGCTCCCTTTTCCCTCCCCAAATGGACATATGGACAGTGCAGGGGACAGAGA  120
             ||||||||||||||||||||||||||||||||||||||||||||||||||||||||||||
Sbjct  6113  CCAAGTGGGGCTCCCTTTTCCCTCCCCAAATGGACATATGGACAGTGCAGGGGACAGAGA  6172

Query  121   CCTTCCATAAAGGAGGGAGCCCCCAAGAGACACTGGTGAAGCCCCAGATACCCAGAGTAC  180
             ||||||||||||||||||||||||||||||||||||||||||||||||||||||||||||
Sbjct  6173  CCTTCCATAAAGGAGGGAGCCCCCAAGAGACACTGGTGAAGCCCCAGATACCCAGAGTAC  6232

Query  181   TGTGCCAAGGAGCCAAGAGTGCTCTGACCAGACCCACTGCTCACTGCCACAGGAAGGAGA  240
             ||||||||||||||||||||||||||||||||||||||||||||||||||||||||||||
Sbjct  6233  TGTGCCAAGGAGCCAAGAGTGCTCTGACCAGACCCACTGCTCACTGCCACAGGAAGGAGA  6292

Query  241   GGTCCTTCCATTGCTTTCCCAACTCATACCTGCTTTTGCCCCATGAATAAAGAGAAGAAA  300
             ||||||||||||||||||||||||||||||||||||||||||||||||||||||||||||
Sbjct  6293  GGTCCTTCCATTGCTTTCCCAACTCATACCTGCTTTTGCCCCATGAATAAAGAGAAGAAA  6352

Query  301   AGATTaaaaaaaaaaaaaaaaaa  323
             |||||||||||||||||||||||
Sbjct  6353  AGATTAAAAAAAAAAAAAAAAAA  6375
```

Fig. S4. *Rtl1* mRNA expression of WT, Pat- and Mat-*Rtl1*Δ.

(A) Quantitative PCR of *Rtl1* in the diaphragm and hindlimb muscles in d16.5 embryo. WT (black, n=8) was adjusted as 1, Pat-*Rtl1*Δ (blue, n=4) and Mat-*Rtl1*Δ (red, n=4). ** $p < 0.01$. Two-tailed Student's t-test was used for the statistical analysis. Error bars indicate stdev. **(B)** The PCR product of Pat-*Rtl1*Δ was cloned, sequenced and confirmed to be the Rtl1 sequence using Standard Nucleotide BLAST (blastn, NCBI nt database).

Development • Supplementary information

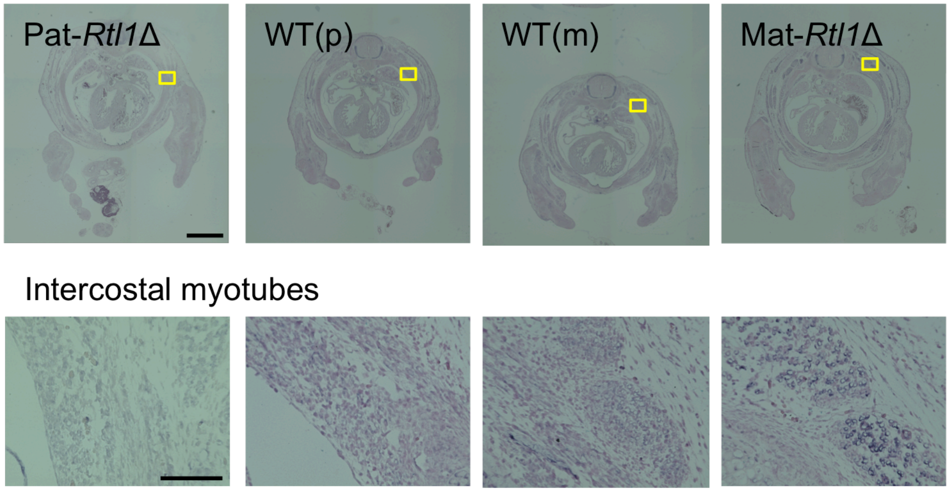

Fig. S5. Immunohistochemical staining of the RTL1 protein in d14.5 embryos. Immunohistochemical staining of a cross-section of the entire embryo (upper column) and a higher magnified view of the intercostal myofibers of the yellow boxes in the upper column (lower column). Pat-*Rtl1*Δ (left), WT (middle) and Mat-*Rtl1*Δ (right). Scale bars, 1mm (upper column), 100 µm (lower column).

Development • Supplementary information

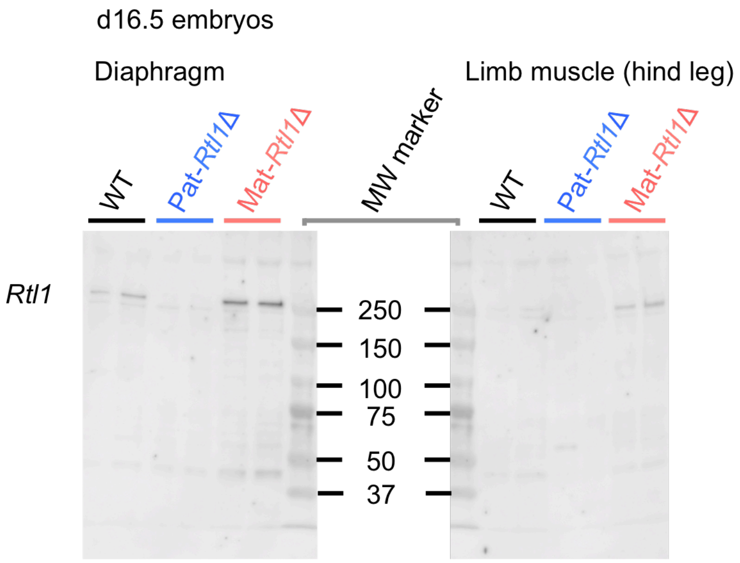

Fig. S6. RTL1 protein expression of WT, Pat- and Mat-*Rtl1*Δ.

Western blotting for RTL1 in the diaphragm and hind limb muscles in d16.5 embryo. WT (left), Pat-*Rtl1*Δ (middle) and Mat-*Rtl1*Δ (right).

Development • Supplementary information

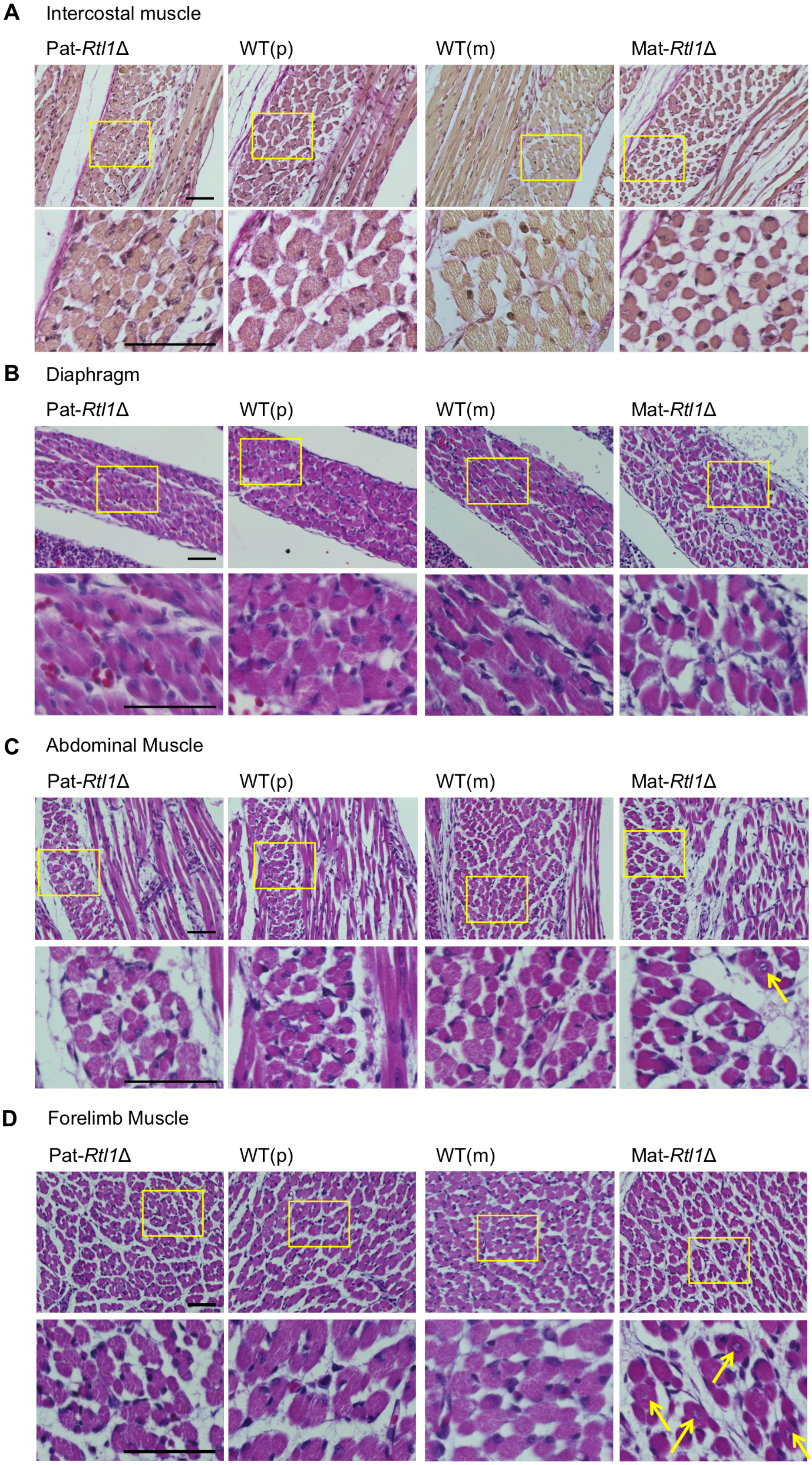

Development • Supplementary information

E

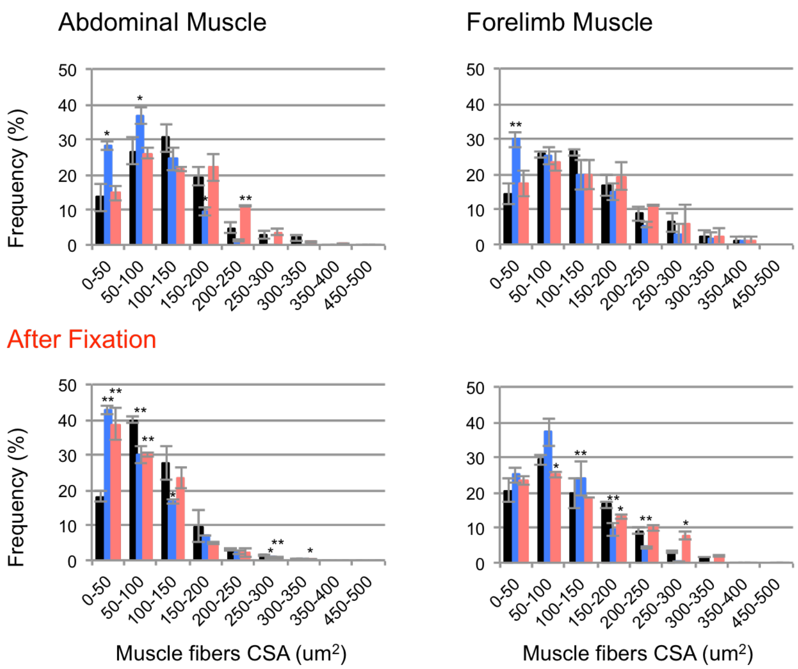

F

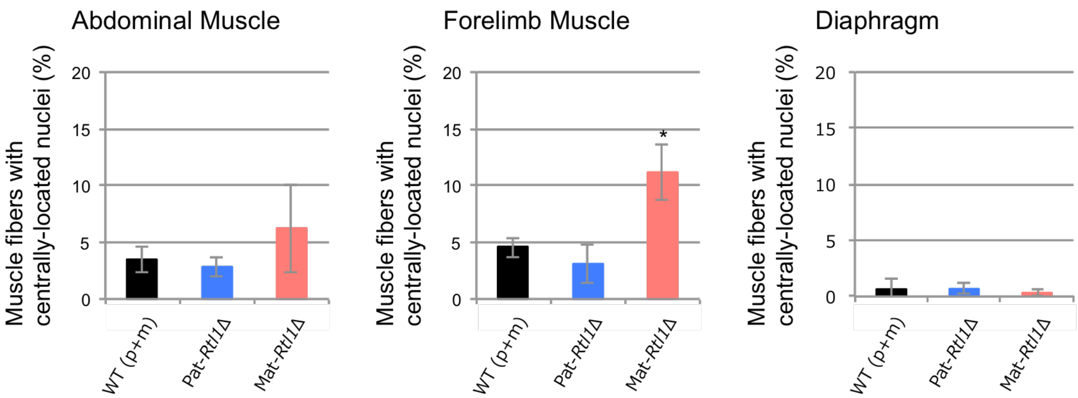

Fig. S7. Histological abnormalities in muscles of Pat- and Mat-*Rtl1*Δ.

(A) Elastica van Gieson (EVG) staining in neonate intercostal muscle tissues of Pat-*Rtl1*Δ (left), WT (middle) and Mat-*Rtl1*Δ (right). **(B-D)** HE staining in neonate intercostal muscle tissues of Pat-*Rtl1*Δ (left), WT (middle) and Mat-*Rtl1*Δ (right). **(B)** Diaphragm muscle (upper column) and a more highly magnified view of the yellow boxes in the upper column (lower column). **(C)** abdominal and **(D)** forelimb muscles. Scale bars, 50 µm. Neonates were fixed in SUPER FIX. **(E)** Distribution of the muscle

fiber size (cross-sectional area: CSA) in WT (black, n=4), Pat-*Rtl1*Δ (blue, n=3) and Mat-*Rtl1*Δ (red, n=3) neonates (non-fixed samples (upper) and fixed samples with SUPER FIX (lower)). **(F)** Proportion of muscle fibers with centrally-located nuclei (arrows in C and D) between WT (black, n=4), Pat-*Rtl1*Δ (blue, n=4) and Mat-*Rtl1*Δ (red, n=4) neonates. Neonates were fixed in SUPER FIX. * $p < 0.05$, ** $p < 0.01$. Two-tailed Student's t-test was used for the statistical analysis. Error bars indicate stdev.

Development • Supplementary information

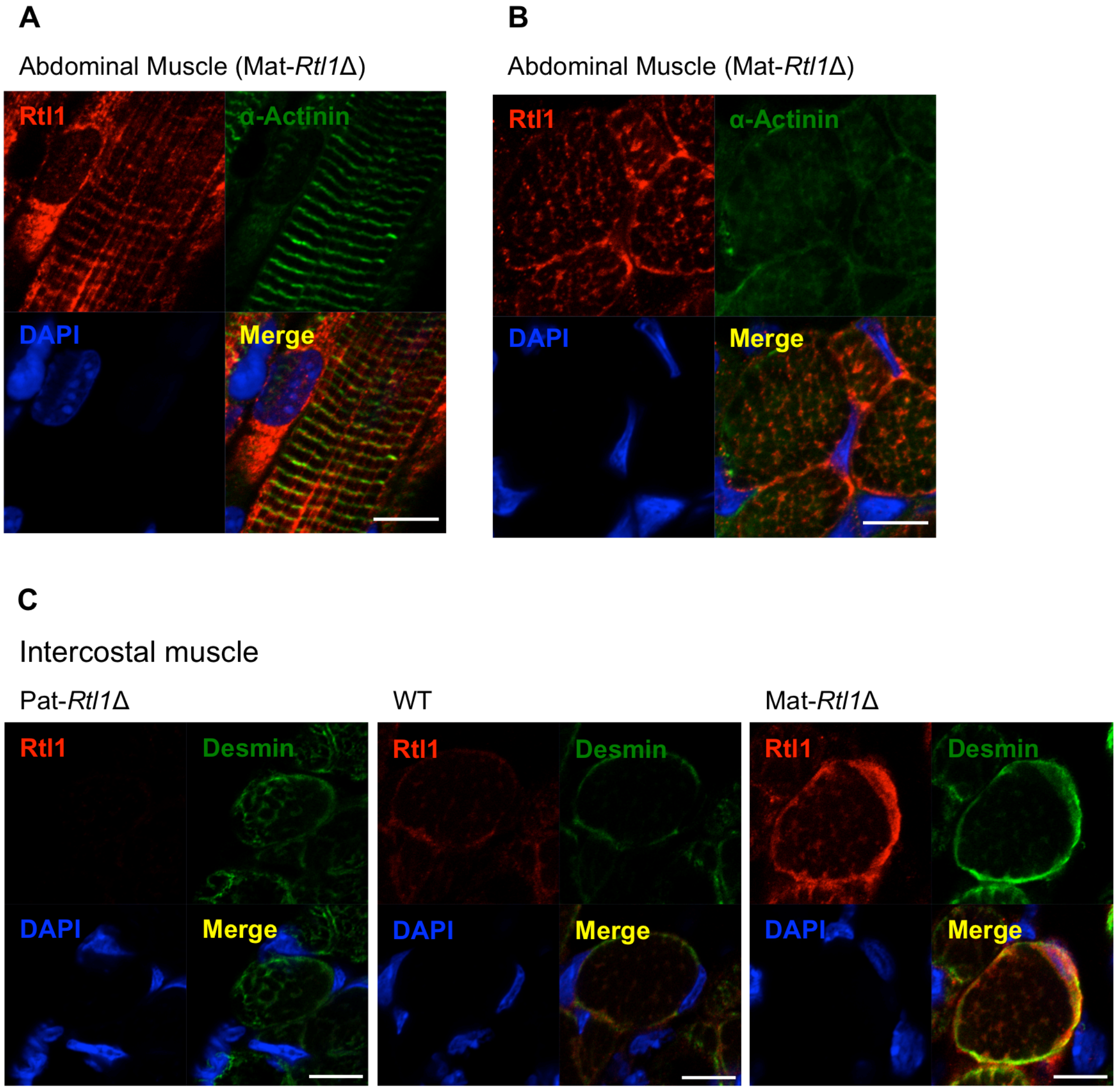

Fig. S8. Expression of *Rtl1* in the neonatal muscle.

(A, B) Immunofluorescence staining of RTL1 protein in the neonatal abdominal muscles from Mat-*Rtl1*Δ mice. Long axis views (A) and cross-sectional views (B) of the muscle fibers of abdominal muscle. Co-immunostaining with RTL1 (red), α-ACTININ (green) and DAPI (blue), and their merged images. **(C)** Cross-sectional views of the muscle fibers of intercostal muscle. Co-immunostaining with RTL1 (red), DESMIN (green) and DAPI (blue), and their merged images. Scale bars, 20 μm. The neonates were not fixed before being embedded in OCT compound.

Development • Supplementary information

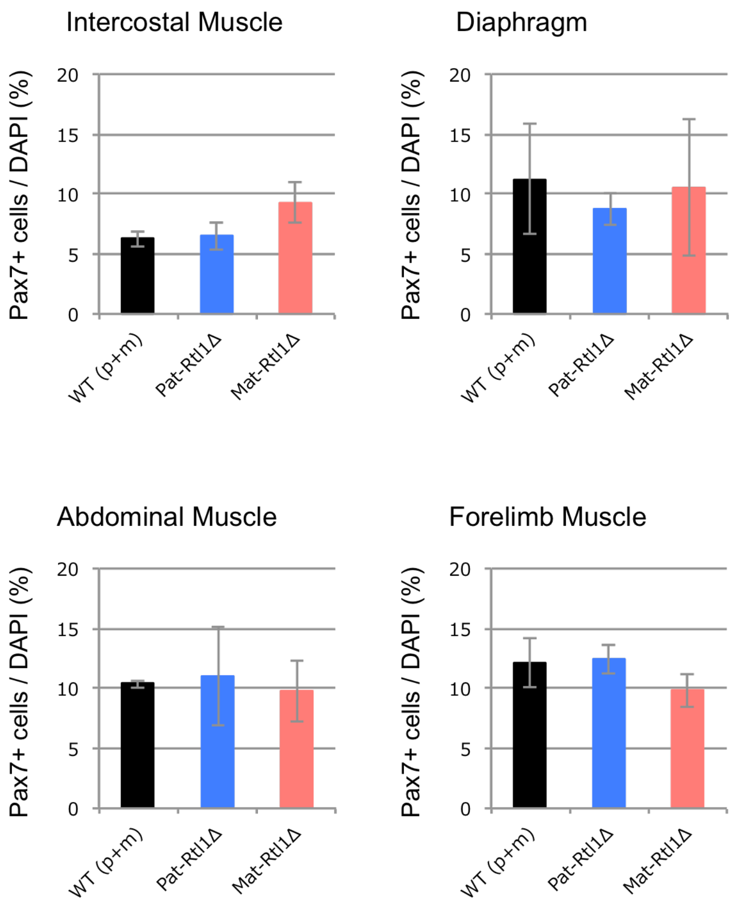

Fig. S9. Proportion of Pax7+ cells in neonate muscles.

Proportion of Pax7-positive cells between WT (black, n=3), Pat-*Rtl1*Δ (blue, n=3) and Mat-*Rtl1*Δ (red, n=3) neonates (non-fixed samples). Two-tailed Student's t-test was used for the statistical analysis. Error bars indicate stdev.

Development • Supplementary information

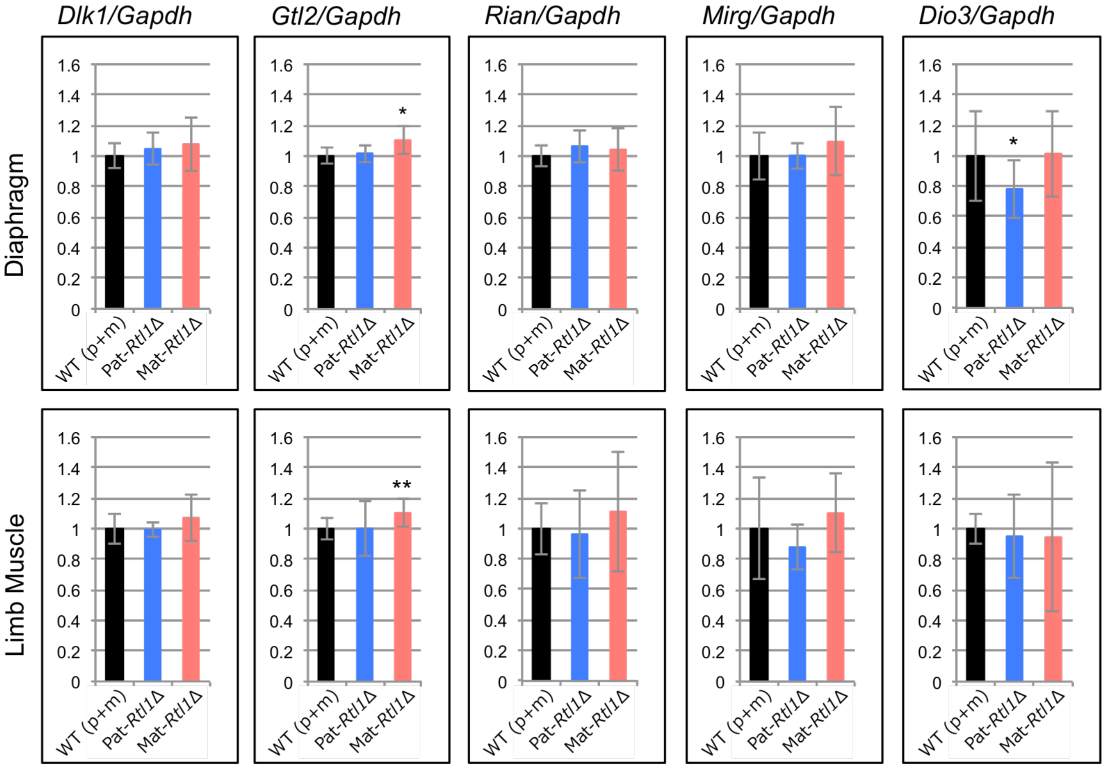

Fig. S10. Expression of imprinted genes in the distal chromosome 12 in mice fetal muscles.

Expression level of the imprinted genes in the WT (black, n=8), Pat-*Rtl1*Δ (blue, n=4) and Mat-*Rtl1*Δ (red, n=4) muscle tissues from d16.5 fetuses as determined by qPCR. *Gapdh* was used as the control. Two-tailed Student's t-test was used for the statistical analysis. Error bars indicate stdev.

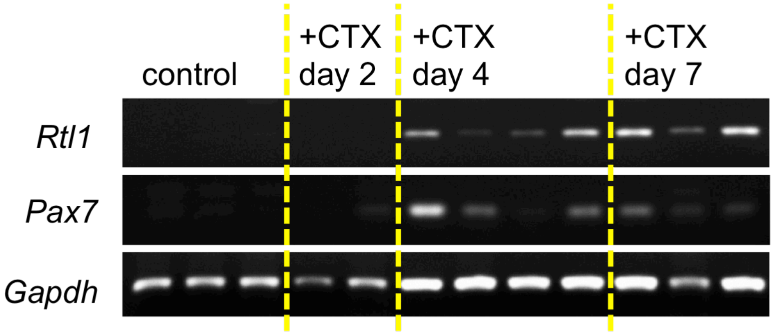

Fig. S11. Expression of *Rtl1* in muscle regeneration process in adult mice after cardiotoxin (CTX) injury.

Expression of *Rtl1* and *Pax7* in intact and regenerating *tibialis anterior* (TA) muscles. The animals were sacrificed on day 2 (n=2), 4 (n=4) and 7 (n=3) after CTX injection. *Gapdh* was used as the control. Results after 35 cycles of amplification were shown.

Development • Supplementary information
