# Supplementary material for: Deficiency and overexpression of Rtl1 in the mouse cause distinct muscle abnormalities related to Temple and Kagami-Ogata syndromes
Source: Development. 2020 Sep 3;147(21):dev185918. doi: 10.1242/dev.185918 (PMC7490516; doi:10.1242/dev.185918)
Supplement: Reviewer comments [file DEV185918_review_history.pdf]

Deficiency and overexpression of *Rtl1* in the mouse cause distinct muscle abnormalities related to Temple and Kagami-Ogata syndromes

Moe Kitazawa, Shinichiro Hayashi, Michihiro Imamura, Shin'ichi Takeda, Yumiko Oishi, Tomoko Kaneko-Ishino and Fumitoshi Ishino

DOI: 10.1242/dev.185918

Editor: Sally Dunwoodie

Review timeline

Original submission: 28 October 2019
Editorial decision: 9 December 2019
First revision received: 11 June 2020
Editorial decision: 1 July 2020
Second revision received: 16 July 2020
Accepted: 20 July 2020

Original submission

First decision letter

MS ID#: DEVELOP/2019/185918

MS TITLE: Lack and overexpression of Peg11/Rtl1 cause distinct muscle abnormalities related to Temple and Kagami-Ogata syndromes, respectively

AUTHORS: Moe Kitazawa, Shinichiro Hayashi, Michihiro Imamura, Yumiko Oishi, Shin'ichi Takeda, Tomoko Kaneko-Ishino, and Fumitoshi Ishino

I have now received all the referees' reports on the above manuscript, and have reached a decision. The referees' comments are appended below, or you can access them online: please go to BenchPress and click on the 'Manuscripts with Decisions' queue in the Author Area.

As you will see, the referees express considerable interest in your work, but have some significant criticisms and recommend a substantial revision of your manuscript before we can consider publication. I encourage you to address all the issues raised by the reviewers excluding the following:

Reviewer 1, Main Point #6: “Unfortunately, this study does not address how Rtl1 controls control muscle development. Could the authors include proteomics data in their study as a first step to address this question? This would be particularly interesting for cell surface proteins (given the observed differential effects on cellular adhesion and of fixation).”

Reviewer 2, Major Concerns #2: “We are left with how PEG11, a retrotransposon, actually regulates muscle development and the mechanism through which it acts.”

Reviewer 2, Comment #6: “The lack of Rtl1 is accompanied by increased proliferation rate of satellites cells (SCs). Did the authors test whether the expression of the other flanking imprinted genes remains unaffected in these in vitro cellular models? Optional – Can we envisage performing rescue experiments by stably integrating Rtl1 transgene in SCs prepared from Pat-KO? If possible, the growth of “rescued SCs” could be used as a phenotypic read-out.”

© 2020. Published by The Company of Biologists under the terms of the Creative Commons Attribution License (https://creativecommons.org/licenses/by/4.0/).

Reviewer 3, Comment #6: “The lack of Rtl1 is accompanied by increased proliferation rate of satellites cells (SCs). Did the authors test whether the expression of the other flanking imprinted genes remains unaffected in these in vitro cellular models? Optional – Can we envisage performing rescue experiments by stably integrating Rtl1 transgene in SCs prepared from Pat-KO? If possible, the growth of “rescued SCs” could be used as a phenotypic read-out.â€¤

Reviewer 3, Comment #10: “Beside its role in muscle, Rtl1 was previously shown to play some roles in placentation, notably in the maintenance of capillaries. Although I am fully aware that the precise molecular mode of action of Rtl1 is beyond the scope of the present manuscript, can the authors speculate on how Rtl1 may exert distinct functions in two different tissues? Is there any common theme that could explain, at least partly, why Rtl1 appears so crucial for both muscular and placental development? Muscle and placenta-derived cells can differentiate into syncytium. As a case in point, it is well established that retroviral protein co-option (e.g. Syncytin-1) plays prominent roles in Syncytiotrophoblasts. Could it be that altered dosage in Rtl1 impacts indirectly on cell fusion processes? This is obviously pure speculation.”

If you are able to revise the manuscript along the lines suggested, which may involve further experiments, I will be happy receive a revised version of the manuscript. Your revised paper will be re-reviewed by one or more of the original referees, and acceptance of your manuscript will depend on your addressing satisfactorily the reviewers' major concerns. Please also note that Development will normally permit only one round of major revision.

Please attend to all of the reviewers' comments and ensure that you clearly highlight all changes made in the revised manuscript. Please avoid using 'Tracked changes' in Word files as these are lost in PDF conversion. I should be grateful if you would also provide a point-by-point response detailing how you have dealt with the points raised by the reviewers in the 'Response to Reviewers' box. If you do not agree with any of their criticisms or suggestions please explain clearly why this is so.

Reviewer 1

Advance summary and potential significance to field

This study explores the imprinted Dlk1-Dio3 domain in the mouse. The authors are particularly interested in the domainÂ’s retrotransposon-derived gene Rrtl1 (Peg11), expressed from the paternal chromosome predominantly. The focus of the study is on a suspected role of this gene in muscle development. This question is important relative to two imprinting disorders that are linked to uniparental disomy of this chromosomal region in humans (Temple Syndrome and the Kagami-Ogata), and in which specific developmental, muscle and skeletal abnormalities are observed.

The main finding of this study is that both paternal Rtl1 deletion in the mouse, leading to a loss of Rtl1 expression, and maternal deletion, leading to increased levels of expression, give distinct muscle phenotypes. These phenotypes are specific to Rtl1 mRNA levels of expression, since the authors did not observe any changes in the RNA expression of the other imprinted genes of the domain as a consequence of the (maternal/paternal) deletion within the Rtl1 gene. Although the phenotype of the Pat-KO was more severe than that of Temple syndrome in humans, the nature of the observed muscle abnormalities showed similarities with this disease. This strongly suggests that also in humans, the muscle phenotype is mostlt caused by loss/overexpression of RTL1 expression. However, in the human situation (Temple syndrome), DLK1 expression is often reduced as well, which has also been linked to muscle abnormalities in different other studies.

The authors also show that purified satellite cells show marked differences in their proliferation rates in vitro as a consequence of the altered expression levels of Rtl1. Furthermore, they make the observation that Pat-Ko myoblasts have a tendency to detach from the culture dish, whereas the Mat-KO muscle fibres have a tendency to shrink upon fixation, decreasing their measured diameter. The study does not further explore what might be the molecular basis of these novel Rtl1-linked cellular phenotypes, and to what extent the observations are relevant to the in vivo situation.

© 2020. Published by The Company of Biologists under the terms of the Creative Commons Attribution License (https://creativecommons.org/licenses/by/4.0/).

Overall, this is a well-performed, but rather descriptive, study that provides evidence for roles of Rtl1 in the muscle, a fiding that is relevant to understand the two imprinting disorders that relate to this conserved imprinted domain. Despite the interest of the presented data to the field, the study could be enhanced by providing initial mechanistic insights, and also by providing a description of the overall phenotype of the Mat-KO and Mat-KO animals, their weight and size.

Comments for the author

Main points:
1) Could the authors please include a brief description of the overall phenotype of their constitutive Mat-KO and Pat-KO mice? Do the observed muscle phenotypes affect relative weight and size of the mice? How do these KO animals compare to earlier KO studies on Rtl1 by others in the field?

2) What is the specificity of the used antiserum directed against Rtl1?
This important question should be assessed by western blotting, including a sample of the Pat-Ko cells.

3) mRNA expression changes often do not translate in significant protein expression levels. Therefore, could the authors assess by western blotting the relative levels of Rtl1 protein expression in the Mat-KO, Pat-KO and WT animals?

4) Figure S4: Where does the residual Rtl1 mRNA expression (about 20%) in the Pat-KO muscles come from? This is unexpected. Is this really a complete KO, and if so, does this transcription originate from somewhere else in the locus? Also for this reason, it will be essential to assess Rtl1 protein expression in the Pat-KO animals.

5) Figure 4C: The pronounced effects on the in vitro cellular proliferation of Satellite Cells (SC), with a strongly increased proliferation in the Pat-KO cells, is most interesting. The authors should explore the relevance of this effect in vivo, particularly to determine whether in the Pat-KO muscle there are more SCs than in the Mat-KO? Also, could the authors determine whether this phenotype correlates with possible alterations in cell cycle regulation?

6) Unfortunately, this study does not address how Rtl1 controls control muscle development. Could the authors include proteomics data in their study as a first step to address this question? This would be particularly interesting for cell surface proteins (given the observed differential effects on cellular adhesion and of fixation).

Minor points:
-Figure 4A: It is unclear what the ‘0’, ‘1’ and ‘3’ indicate. Please, explain this in the legend.

-The title is somewhat misleading, and could be changed as follows: Lack and overexpression of Peg11/Rtl1 in the mouse cause muscle abnormalities observed in Temple and Kagami-Ogata syndromes.

-Line 44: there are many cases of TS and KOS14 that are not caused by disomy.
Please, change the sentence.

-Throughout the text, could the authors please choose one name, either Rtl1 (used by many in the field) or Peg11 (the name given by the authors). What is the name recognised by the nomenclature committee?

-Line 243: It has not been proven that loss and overproduction of RTL1 leads to TS and KOS14, respectively. Not in this study, and also not in earlier human studies. Please, change.

© 2020. Published by The Company of Biologists under the terms of the Creative Commons Attribution License (https://creativecommons.org/licenses/by/4.0/).

Reviewer 2

Advance summary and potential significance to field

The authors the role of Peg11 in muscle cells. They found that deletion of the paternal Peg11 deletion and maternal Peg11 deletion (deletes maternally expressed microRNAs, resulting in Peg11 overexpression) produced neonatal skeletal muscle defects and abnormalities in satellite cell proliferation. They conclude that deletion of the paternal Peg11 allele and deletion of the maternal Peg11as allele represent models for Temple and Kagami-Ogata syndromes.

Comments for the author

In the manuscript entitle “Lack and overexpression of Peg11/Rt11 cause distinct muscle abnormalities related to Temple and Kagami-Ogata syndromes, respectively”, Kitazawa and colleagues continue their assessment of loss and overexpression of the imprinted Peg11 gene. In this manuscript, they specifically examined the role of *Peg11* in muscle cells. They found that deletion of the paternal Peg11 deletion and maternal Peg11 deletion (deletes maternally expressed microRNAs, resulting in Peg11 overexpression produced neonatal skeletal muscle defects and abnormalities in satellite cell proliferation. They conclude that deletion of the paternal Peg11 allele and deletion of the maternal Peg11as allele represent models for Temple and Kagami-Ogata syndromes. The authors make contributions to our understanding of Peg11’s role in muscle cells. However, there a several concerns that need to be addressed.

Major concerns
Abstract, lines 31-35. The authors state that “we have demonstrated that Peg11/Rtl1 also plays an important role in fetal/neonatal skeletal muscle development as a novel Z-disc-interacting component”. While the authors show PEG11 staining at Desmin and Actinin Z-discs, there is no genetic or biochemical data to support that this protein is a Z-disc-interacting protein.
We are left with how PEG11, a retrotransposon, actually regulates muscle development and the mechanism through which it acts.
Abstract and discussion. The authors present the idea that Peg11 evolved as an adaptation to eutherian viviparous reproduction. This is pure speculation, detracts from the primary story, and lacks experimental data. I strongly suggest that the evolution sections be removed from the manuscript.
Gene deletion name. The authors refer to the deletions as Pat-KO and Mat-KO. This is too vague and confusing. In addition, the accepted gene name for *Peg11* is *Rtl1* and should be used, as the authors described in their original paper (Sekita et al., 2008; not necessary to state *Peg11*/*Rtl1* throughout the manuscript; similar comments for the Peg and Meg genes in Fig S8 for using the accepted names). The paternal deletion should be referred to as Pat-*Rtl1*-KO or more accurately as Pat-$Rtl1^{\Delta}$. There is no accepted gene name for *Rtl1* antisense gene and thus can be referred to as *Rtl1as* or *Peg11as*. The maternal deletion should be called Mat-$Rtl1^{\Delta}$.
Genetic models: Although the deletion mice were described in another manuscript, they warrant an explanation to understand this manuscript. I suggest that the results section start with the genetic models and should include the follow information: The *Rtl1* deletion removes most of the *Rtl1* gene, as well as microRNA encoded on the antisense strand. On the paternal allele, the retrotransposon *Rtl1*gene is expressed, while the *Rtl1as* gene is silent. A paternal-inherited *Rtl1* deletion results in loss of *Rtl1* expression. On maternal allele, *Rtl1as* is expressed and *Rtl1* is silent. The *Rtl1as* transcript produces microRNAs that degrade the *Rtl1* transcript. Thus, a maternally inherited *Rtl1* deletion result in loss of maternal microRNA expression, leading to overexpression of paternal *Rtl1* allele. The authors should then include their data on other imprinted genes in the *Gtl2* region, showing that *Rtl1* and *Rtl1as* deletions, for the most part, did not affect expression of other genes in the imprinted domain. Thus, any affects observed in deletion mice will allow phenotypes to be assigned to *Rtl1* and *Rtl1as* genes. I suggest combining Fig S1 and S2, and Fig S8 should become Fig S2.
Since the Rtl1 deletion removed microRNAs, can the authors solely attribute phenotypes to Rtl1 overexpression and not consider other microRNA targets?
Lines 159-184, this section on evolution should be deleted. While the authors summarize the findings of papers on phenotypes in humans in Table S1-S3, the data was not produced by the authors. This section is also presented in discussion, where it belongs.

© 2020. Published by The Company of Biologists under the terms of the Creative Commons Attribution License (https://creativecommons.org/licenses/by/4.0/).

Minor concerns

1. I would suggest that the authors indicate in the text where results are statistically different.
2. Do the “*” in figures, represent statistical differences compared to controls?
3. Protein name should be all Caps, i.e. RTL1
4. Line 81, the authors state “expression was rapidly reduced after birth”. A decreased in expression over a 15 day period is not rapid.
5. Line 98, how did the authors determine that muscle fibers became detached from the extracellular matrix? Either ECM staining is required or a greater explanation is needed if this observation is inferred from the literature.
6. Line 152, the authors state that “SCs evidently displayed weak or low intensity”. Weak or low intensity of what?
7. Fig 159, change “Tample” to “Temple”.
8. Fig 2A and 2B, add lines to direct readers to look at “thinner” and “larger” muscle fibers.
9. Fig 2A and 2B would benefit readers if there were made larger.
10. Fig 2C, to what are the yellow arrows pointing? Add this information to the figure legend. Normal nuclei should be indicated in the WT panel.
11. Fig S6A, where nuclei not centrally located in the diaphragm? Fig 6E, was the frequency of centrally located nuclei in the abdominal muscle significantly different?
12. Fig 2D and Fig S6D, explain CSA in figure legend.
13. Fig 4G, Are the SCs thinner with Pat-KO and thicker with Mat-KO?
14. Figure legends S1 and S2 would benefit from more explanation.
15. Table S1 legend, change “syndrom” to “syndrome”
16. Table S1, is the lethality in mice compared to humans Temple syndrome because of medical interventions?
17. Table S1, should it be “no data reported but could be observed (add ref)”?
18. Table S1 what is “SI” in last line?
19. Table S2 and S3, at the bottom it states that “Abnormalities observed in abdomen or thorax”, should this be part of the titlea?
20. Table S3, at bottom it states that “(): respiratory failure”. Was this supposed to be part of lower table?
21. Should there be similar tables for Temple Syndrome?

<u>Reviewer 3</u>

Advance summary and potential significance to field

Deficiencies in gene expression at the imprinted DlK1-Dio3 chromosomal region are genetically associated with two rare human disorders: the Temple and Kagamai-Ogata syndromes (matUPD14 and patUD14, respectively). Through the use of two genetically-modified mouse models (Rtl1 knockout and Rtl1 overexpression), the authors show that Rtl1 a LTR retrotransposon-derived gene - plays some roles in foetal and neonatal muscle development. Moreover, the authors discuss its potential involvement in muscle symptoms that feature the Temple and Kagami-Ogata syndromes. Finally, the evolutionary impact of the recently-evolved Rtl1 gene in eutherian mammals is highlighted. The manuscript is concise and relatively well written, with most of the interpretations supported by experimental data. Although the authors do not provide molecular insights into the mode of action of Rtl1, I found this work very instructive. I have no major criticism. Prior to consideration for publication, however, I would appreciate that the authors address my specific (relatively minor) comments which are listed below.

Comments for the author

#1 – In order to facilitate the reading of the manuscript, I recommend that the authors incorporate Figure S1 in the full manuscript. Indeed, this will render more explicit the fact that overexpression of the paternally-expressed Rtl1 gene is due to the lack of several maternally-expressed miRNA genes (see below #3). A schematic representation of breeding strategies to generate KO-Pat and KO-Mat individuals, including their WT littermates, must also be shown (see below #7).

© 2020. Published by The Company of Biologists under the terms of the Creative Commons Attribution License (https://creativecommons.org/licenses/by/4.0/).

#2 – The main message delivered by the authors is the notion that altered Rtl1 expression may account for muscle abnormalities described in Temple and Kagami-Ogata syndromes. Accordingly, I propose that Table S1 also moves to the full paper. Again, this will greatly facilitate the comparison between mouse and human studies.

#3 - In Mat-KO mice, elevated expression of Rtl1 is due to the lack of expression of the maternally-expressed miR-136/miR-431 cluster. Can the authors exclude the possibility that muscle abnormalities seen in Mat-KO mice originate from mis-expression of additional mRNAs targeted by these peculiar antisense miRNAs? In other words, can we rule out the possibility that the miR-136/miR-431 cluster targets other mRNAs for silencing, and in doing so, contributes to muscle defects through Rtl1-independent manner?

#4 – Lane 97 – I am not sure that I understand correctly the technical importance, if any, of using fixation with SUPER FIX, and its potential link with the fact that "Mat-KO mice displayed severe shrinkage …" Could you please comment ?

#5 - Fig.3A – Why DAPI staining is shown in red, and not blue as classically used, including in the flanking panel 3B. To further demonstrate the specificity of the detected IF signals, it might also be appropriated to use Pat-KO samples as negative controls.

#6 – The lack of Rtl1 is accompanied by increased proliferation rate of satellites cells (SCs). Did the authors test whether the expression of the other flanking imprinted genes remains unaffected in these in vitro cellular models? Optional – Can we envisage performing rescue experiments by stably integrating Rtl1 transgene in SCs prepared from Pat-KO? If possible, the growth of "rescued SCs" could be used as a phenotypic read-out.

#7 – Throughout the text, the authors employ the term WT. Does it refer to WT littermates? If yes, the authors should then compare, in my view, Pat-KO and Mat-KO to their respective WT littermates. Accordingly, two WT controls should be shown.

#8 – Lane 159 - Title "Temple" and not "Tample"

#9 – Lane 212"Therefore, it is clear that combined and differential involvement of …" The authors may also include additional references indicating that Dlk1-Dio3-derived miRNAs play some roles in muscular system and, in doing so, may thus contribute to phenotype described in Temple and Kagami-Ogata syndromes. Among others are Wüst et al (2018) Cell Metabol; Castel et al (2018) Sci Rep., Gao et al (2015) PNAS

#10 – Beside its role in muscle, Rtl1 was previously shown to play some roles in placentation, notably in the maintenance of capillaries. Although I am fully aware that the precise molecular mode of action of Rtl1 is beyond the scope of the present manuscript, can the authors speculate on how Rtl1 may exert distinct functions in two different tissues? Is there any common theme that could explain, at least partly, why Rtl1 appears so crucial for both muscular and placental development? Muscle and placenta-derived cells can differentiate into syncytium. As a case in point, it is well established that retroviral protein co-option (e.g. Syncytin-1) plays prominent roles in Syncytiotrophoblasts. Could it be that altered dosage in Rtl1 impacts indirectly on cell fusion processes? This is obviously pure speculation.

First revision

Author response to reviewers' comments

Thank you very much for all the reviewer's valuable comments that help us improve the revised manuscript. We addressed them as far as possible and really hope that the changes we made satisfy the requests from the reviewers.

© 2020. Published by The Company of Biologists under the terms of the Creative Commons Attribution License (https://creativecommons.org/licenses/by/4.0/).

Reviewer 1 Comments for the Author:
Main points:
1) Could the authors please include a brief description of the overall phenotype of their constitutive Mat-KO and Pat-KO mice?

Thank you very much for your suggestion and we have received similar suggestions from other reviewers. We added a detailed description of the phenotype of Pat- and Mat-Rtl1Δ in the introduction section.

Do the observed muscle phenotypes affect relative weight and size of the mice?

There may be a correlation between the weight and muscle fiber size in the case of Pat-Rtl1Δ because the Pat-Rtl1Δ mice are born smaller and have less fat, but the Mat-Rtl1Δ mice do not exhibit any significant weight difference compared with the WT.

How do these KO animals compare to earlier KO studies on Rtl1 by others in the field?

To the best of our knowledge, there are very few reports on Rtl1 KO by other researchers. There is only the report by Ito et al., 2015 (we are collaborators in that work), which demonstrated that mice with approximately the latter half of Rtl1 deleted exhibited a mild phenotype compare to our models. ΔRtl1 (paternal transmission) and ΔmiR-127 (maternal transmission) did not exhibit lethality, but the former did exhibit pre- and postnatal growth retardation and the latter had mild placentomegaly, consistent with our previous work (Sekita et al. 2006). Recently, during the submission of this manuscript, another paper by Loo et al. in 2019 reported that RTL1 is involved in muscle regeneration under the control of the LINC complex component SUN1. We have cited both references in the revised version. The mice with chromosome 12 disomy are comparable (Geordiades et al., 2000) and are discussed in Table 1.

2) What is the specificity of the used antiserum directed against Rtl1? This important question should be assessed by western blotting, including a sample of the Pat-Ko cells.
3) mRNA expression changes often do not translate in significant protein expression levels. Therefore, could the authors assess by western blotting the relative levels of Rtl1 protein expression in the Mat-KO, Pat-KO and WT animals?

We agree that this is a very important point. In our Western blotting experiment, the RTL1 protein was detected in the d16.5 fetal diaphragm and hind limb muscle as a 250 kDa band concomitantly with a minor, smaller band around 200 kDa (Supplementary material Fig. S6). Compared to the WT, the amount of RTL1 expression is clearly higher in Mat-Rtl1Δ. In Pat-Rtl1Δ, the major band is almost invisible but the smaller, minor band is slightly visible. We think this consistent with the Rtl1 mRNA expression data shown in Fig. S4. We cloned and sequenced the PCR product of Pat-Rtl1Δ and confirmed that this product was actually from Rtl1 mRNA. We think that this is due to leaky expression from a maternal allele but we don't know why only a smaller minor band was detected, although there are several possibilities, such as translation from the second or third ATG, the presence of a differently spliced form, and/or a different transcription start site was used in the usually repressed maternal alleles.

4) Figure S4: Where does the residual Rtl1 mRNA expression (about 20%) in the Pat-KO muscles come from? This is unexpected. Is this really a complete KO, and if so, does this transcription originate from somewhere else in the locus? Also for this reason, it will be essential to assess Rtl1 protein expression in the Pat-KO animals.

We confirmed that a small amount of the Rtl1 mRNA in Pat-Rtl1Δ was actually from Rtl1. The PCR product was cloned and sequenced, and confirmed to be the Rtl1 sequence. It is thought that the residual Rtl1 mRNA expression in the Pat-Rtl1Δ muscles may be expressed from a maternal allele (leaky expression).

5) Figure 4C: The pronounced effects on the in vitro cellular proliferation of Satellite Cells (SC), with a strongly increased proliferation in the Pat-KO cells, is most interesting. The authors should explore the relevance of this effect in vivo, particularly to determine whether in the Pat-KO muscle

© 2020. Published by The Company of Biologists under the terms of the Creative Commons Attribution License (https://creativecommons.org/licenses/by/4.0/).

there are more SCs than in the Mat-KO? Also, could the authors determine whether this phenotype correlates with possible alterations in cell cycle regulation?

We counted the number of Pax7+ satellite cells in muscle in the WT, Pat-Rtl1Δ and Mat-Rtl1Δ, but did not obtain a positive result: there was no significant difference in the number of Pax7+ satellite cells. We were unable to investigate cell cycle regulation in vivo because of a limitation of the animal facility (RI usage in animals).

6) Unfortunately, this study does not address how Rtl1 controls control muscle development. Could the authors include proteomics data in their study as a first step to address this question? This would be particularly interesting for cell surface proteins (given the observed differential effects on cellular adhesion and of fixation).

We agree that this is very interesting in terms of muscle development in eutherians. However, the purpose of this study was to identify the cause of Kagami-Ogata syndrome and Temple syndrome. Therefore, we would like to address this point in a subsequent study.

Minor points:
-Figure 4A: It is unclear what the 0, 1 and 3 indicate. Please, explain this in the legend.

We added "Day" in the figure and the sentence SCs during proliferation (day 0) and after inducing differentiation (1 and 3 days) are shown" in the figure legend.

-The title is somewhat misleading, and could be changed as follows: Lack and overexpression of Peg11/Rtl1 in the mouse cause muscle abnormalities observed in Temple and Kagami-Ogata syndromes.

We added "in the mouse" in the title.

-Line 44: there are many cases of TS and KOS14 that are not caused by disomy. Please, change the sentence.

Thank you very much for this comment. It is true that there are cases caused by epimutations or microdeletions in this imprinted region (Kagami et al., 2008). We have accordingly changed the sentence to "Temple and Kagami-Ogata syndromes are caused by maternal and paternal disomy of chromosome 14…". We have also added a comment and discuss epimutations and microdeletions in lines 275-284.

-Throughout the text, could the authors please choose one name, either Rtl1 (used by many in the field) or Peg11 (the name given by the authors). What is the name recognised by the nomenclature committee?

We unified the terminology to Rtl1. We also added the sentence in lines 47-50 "Retrotransposon Gag like 1 (RTL1) was first identified as PEG11 in a DLK1-DIO3 imprinted region in sheep (Charlier et al., 2001), and later was formally renamed RTL1 as one of the genes derived from a suchi-ichi-related LTR retrotransposon in humans and mice."

-Line 243: It has not been proven that loss and overproduction of RTL1 leads to TS and KOS14, respectively. Not in this study, and also not in earlier human studies. Please, change.

Thank you for your suggestion. We changed the sentence to "Both the loss and overproduction of RTL1 affect the muscle phenotypes of the Temple and Kagami-Ogata syndrome, respectively."

Reviewer 2 Comments for the Author:
Major concerns
1. Abstract, lines 31-35. The authors state that "we have demonstrated that Peg11/Rtl1 also plays an important role in fetal/neonatal skeletal muscle development as a novel Z-disc-interacting component". While the authors show PEG11 staining at Desmin and Actinin Z-discs, there is no genetic or biochemical data to support that this protein is a Z-disc-interacting protein.

© 2020. Published by The Company of Biologists under the terms of the Creative Commons Attribution License (https://creativecommons.org/licenses/by/4.0/).

We agree with this comment and have deleted the sentence containing the phrase as a novel Z-disc-interacting component from the abstract and inserted a sentence in the text that reads The RTl1 protein is localized near the Z-disc in fetal/neonatal muscle fibers close to the DESMIN protein.

2. We are left with how PEG11, a retrotransposon, actually regulates muscle development and the mechanism through which it acts.

It is a very important question and we really want to know the answer, but it is a very difficult challenge. All that we know at present is that REG11(RTL1) has a retroviral type of protease domain. We are currently engaged in an effort to make KI mice that have an inactive protease motif. We hope this effort will succeed and provide a means to solve the question.

3. Abstract and discussion. The authors present the idea that Peg11 evolved as an adaptation to eutherian viviparous reproduction. This is pure speculation, detracts from the primary story, and lacks experimental data. I strongly suggest that the evolution sections be removed from the manuscript.

We agree with this comment. We deleted the sentence from the abstract and replaced it with the following. “This is the first example of an LTR retrotransposon-derived gene specific to eutherians contributing to the evolution of eutherian skeletal muscle.” Moreover, the most interesting and important point of RTL1 (PEG11) is that it is a eutherian-specific acquired gene, so it should be discussed from the point of view of evolution. In the discussion, we clearly indicate this to be speculation and discuss it as shown below:
In lines 370-375 “We speculate that it also plays a role as a suppressor of DESMIN to prevent fast and vigorous muscle movement in the fetal/neonatal stages by interacting or interfering with DESMIN. This speculation is supported by the fact that Desmin KO mice exhibit no gross abnormality in the fetal/neonatal periods, but rather, exhibit severe muscle defects around 2 weeks after birth (Milner et al., 1996; Li et al., 1997).” And in lines 379-383, “This view appears to be consistent with the fact that human babies and mouse pups exhibit slow and weak muscle movement just before and after birth. This kind of inhibitory function seems advantageous for both mothers and fetuses/neonates to ensure safety for pregnancy and child-rearing, implying that it was one of important adaptations to the mammalian viviparaous reproduction system.”

4. Gene deletion name. The authors refer to the deletions as Pat-KO and Mat-KO. This is too vague and confusing. In addition, the accepted gene name for Peg11 is Rtl1 and should be used, as the authors described in their original paper (Sekita et al., 2008; not necessary to state Peg11/Rtl1 throughout the manuscript; similar comments for the Peg and Meg genes in Fig S8 for using the accepted names). The paternal deletion should be referred to as Pat-Rtl1-KO or more accurately as Pat-Rtl1Δ. There is no accepted gene name for Rtl1 antisense gene and thus can be referred to as Rtl1as or Peg11as. The maternal deletion should be called Mat-Rtl1Δ.

Thank you very much for this critical suggestion. We have changed the terms to Pat-Rtl1Δ and Mat-Rtl1Δ throughout the text.

5. Genetic models: Although the deletion mice were described in another manuscript, they warrant an explanation to understand this manuscript. I suggest that the results section start with the genetic models and should include the follow information: The Rtl1 deletion removes most of the Rtl1 gene, as well as microRNA encoded on the antisense strand. On the paternal allele, the retrotransposon Rtl1gene is expressed, while the Rtl1as gene is silent. A paternal-inherited Rtl1 deletion results in loss of Rtl1 expression. On maternal allele, Rtl1as is expressed and Rtl1 is silent. The Rtl1as transcript produces microRNAs that degrade the Rtl1 transcript. Thus, a maternally inherited Rtl1 deletion result in loss of maternal microRNA expression, leading to overexpression of paternal Rtl1 allele. The authors should then include their data on other imprinted genes in the Gtl2 region, showing that Rtl1 and Rtl1as deletions, for the most part, did not affect expression of other genes in the imprinted domain. Thus, any affects observed in deletion mice will allow phenotypes to be assigned to Rtl1 and Rtl1as genes. I suggest combining Fig S1 and S2, and Fig S8 should become Fig S2.

© 2020. Published by The Company of Biologists under the terms of the Creative Commons Attribution License (https://creativecommons.org/licenses/by/4.0/).

Thank you very much for your helpful suggestion. We have added a detailed description of Pat-Rtl1Δ and Mat-Rtl1Δ in the introduction section and put a section called Genetic models" in the first part of the Results section. We hope that these changes satisfy your request.

6. Since the Rtl1 deletion removed microRNAs, can the authors solely attribute phenotypes to Rtl1 overexpression and not consider other microRNA targets?

This is a most critical point. As you know, the possibility of other target genes cannot be completely ruled out. There are many reports suggesting miRNAs in the DLK1 and DIO3 imprinted regions. Moreover, to the best of our knowledge, the relationship between the miRNAs encoded by the Rtlas and muscle development was formally suggested by Wu et al., 2015. We discuss this point in the discussion, as shown below:
Lines 285-299, "This study provides strong support that the loss and overproduction of human RTL1 are the major cause of the muscle symptoms observed in Temple syndrome and Kagami-Ogata syndrome, respectively. We cannot rule out the possibility that some of the miRNA(s) in the miR-127/miR-136 cluster in Rtl1as have different target gene(s) other than RTL1 and are involved in muscle development. For example, it is reported that miR-431 in the miR-127/miR-136 cluster promotes myogenic differentiation by targeting Pax7 (Wu et al., 2014). The authors showed that miR-431 regulates the Pax7 levels during muscle development and regeneration using two miR-431 TG strains of mice (4- and 20-fold overexpression of miR-431, respectively), but there is no difference in Rtl1 expression in the skeletal muscle of miR-431 TG and WT mice, concluding that miR-431 regulates myogenic differentiation independently of Rtl1. However, in our models, the Pax7 expression level was not affected in the Mat-Rtl1Δ SCs (without miR-431 and other 5 miRNAs), while it was up-regulated in the Pat-Rtl1Δ SCs (Fig. 4B), indicating that the phenotypes we observed in this study are independent of the miR-431-Pax7 pathway."

7. Lines 159-184, this section on evolution should be deleted. While the authors summarize the findings of papers on phenotypes in humans in Table S1-S3, the data was not produced by the authors. This section is also presented in discussion, where it belongs.

We could not identify the evolutionary statement in Lines 159-184 but reduced the opinion throughout the text. Concerning to the Table S1, the opinion on this point differs among the reviewers. The other two reviewers suggest the data should include Table 1 in the text for a detailed comparison. Therefore, we moved the Table S1 to Table 1.

Minor concerns
1. I would suggest that the authors indicate in the text where results are statistically different.

We have added "significantly" with p values in the text wherever possible.

2. Do the "*" in figures, represent statistical differences compared to controls?

Yes. We explained this in the figure legends.

3.Protein name should be all Caps, i.e. RTL1

Thank you for your comment. We changed it to "RTL1".

4. Line 81, the authors state "expression was rapidly reduced after birth". A decreased in expression over a 15 day period is not rapid.

We agree with this comment. We have changed it to "gradually".

5. Line 98, how did the authors determine that muscle fibers became detached from the extracellular matrix? Either ECM staining is required or a greater explanation is needed if this observation is inferred from the literature.

We added the data from Elastica Van Gieson (EVG) staining in the Supplementary materials Fig. 7A. The separation of the extracellular matrix (collagen) and muscle fibers, both stained in red, can be

© 2020. Published by The Company of Biologists under the terms of the Creative Commons Attribution License (https://creativecommons.org/licenses/by/4.0/).

clearly seen in the case of Mat-Rtl1Δ, indicating the muscle fibers of Mat-Rtl1Δ were detached from the extracellular matrix by severe shrinkage.

6. Line 152, the authors state that SCs evidently displayed weak or low intensity. Weak or low intensity of what?

Thank you for your comment. We changed this part to "..weak or low structural strength of myoblast cells".

7. Fig 159, change 'Tample' to 'Temple'.

We changed this to 'Temple'. Thank you.

8. Fig 2A and 2B, add lines to direct readers to look at "thinner" and "larger" muscle fibers.

9. Fig 2A and 2B would benefit readers if there were made larger.

We added a higher magnified view, and indicated the thinner muscle fibers in Pat-Rtl1Δ with arrows and the large muscle fibers in Mat-Rtl1Δ with arrowheads.

10. Fig 2C, to what are the yellow arrows pointing? Add this information to the figure legend. Normal nuclei should be indicated in the WT panel.

We added the following sentence to the figure legend 2C 'The arrowheads in the WT column indicate normal nuclei and the arrows in the Mat-Rtl1Δ column indicate the muscle fibers with centrally-located nuclei.'

11. Fig S6A, where nuclei not centrally located in the diaphragm? Fig 6E, was the frequency of centrally located nuclei in the abdominal muscle significantly different?

Thank you for your comment. Actually, neither abdominal muscle nor the diaphragm exhibited any significant difference. We added the data on the diaphragm (i.e. the muscle fibers with the centrally located nuclei) to the Supplementary materials Fig. S7F.

12. Fig 2D and Fig S6D, explain CSA in figure legend.

We added an explanation of the "CSA: cross-sectional area" in the figure legends.

13. Fig 4G, Are the SCs thinner with Pat-KO and thicker with Mat-KO?

We did not see any difference in the proliferating SCs. It was difficult for us to measure the diameter of the myoblasts differentiated from the SCs.

14. Figure legends S1 and S2 would benefit from more explanation.

We added further explanation to the figure legends.

15. Table S1 legend, change 'syndrom' to 'syndrome'

Thank you. We amended it to "syndrome.

16. Table S1, is the lethality in mice compared to humans Temple syndrome because of medical interventions?

We added the table of the Temple syndrome according to the suggestion of reviewer 3. These are less lethal in humans like F1 mice. We also added an explanation of this in the introduction because the lethality of Pat-Rtl1Δ mice was totally dependent on the genetic background, that is, all of those on the pure B6 background died, but none on the F1 background died, such as 129/B6 or DBA2/B6. Therefore, we don' t think medical intervention is relevant in terms of lethality.

© 2020. Published by The Company of Biologists under the terms of the Creative Commons Attribution License (https://creativecommons.org/licenses/by/4.0/).

17. Table S1, should it be no data reported but could be observed (add ref)?

Thank you very much. We amended this according to your advice.

18. Table S1 what is SI in last line?

That is a mistake, so we deleted it.

19. Table S2 and S3, at the bottom it states that 'Abnormalities observed in abdomen or thorax', should this be part of the titlea?

Thank you for your comment. We changed the titles and added an explanation of Tables S1 and S2.

20. Table S3, at bottom it states that (): respiratory failure". Was this supposed to be part of lower table?

We agree it is not easy to understand. We added an explanation of the second column in the Table S3 legend. (Table S2 in this revised version).

21. Should there be similar tables for Temple Syndrome?

Thank you for your suggestion. We added a table for the Temple syndrome.

Reviewer 3 Advance Summary and Potential Significance to Field:
Reviewer 3 Comments for the Author:
#1 – In order to facilitate the reading of the manuscript, I recommend that the authors incorporate Figure S1 in the full manuscript. Indeed, this will render more explicit the fact that overexpression of the paternally-expressed Rtl1 gene is due to the lack of several maternally-expressed miRNA genes (see below #3). A schematic representation of breeding strategies to generate KO-Pat and KO-Mat individuals, including their WT littermates, must also be shown (see below #7).

Thank you very much for these comments. We added a section on 'Genetic models' at the beginning of the Results section and made a schematic representation of the breeding strategies used to generate Pat-Rtl1Δ and Mat-RtlΔ in the Supplementary materials Fig. S2C. We also added an explanation of Pat-RtlΔ and Mat-Rtl1Δ in the Introduction.

#2 – The main message delivered by the authors is the notion that altered Rtl1 expression may account for muscle abnormalities described in Temple and Kagami-Ogata syndromes. Accordingly, I propose that Table S1 also moves to the full paper. Again, this will greatly facilitate the comparison between mouse and human studies.

Thank you for your advice. We moved Table S1 to Table 1.

#3 - In Mat-KO mice, elevated expression of Rtl1 is due to the lack of expression of the maternally-expressed miR-136/miR-431 cluster. Can the authors exclude the possibility that muscle abnormalities seen in Mat-KO mice originate from mis-expression of additional mRNAs targeted by these peculiar antisense miRNAs? In other words, can we rule out the possibility that the miR-136/miR-431 cluster targets other mRNAs for silencing, and in doing so, contributes to muscle defects through Rtl1-independent manner?
We agree that this is the most important critical comment on our work.

Honestly, the possibility of other target genes cannot be completely ruled out. There is only one reference (Wu et al., 2015) of which we are aware, and it indicates that miR-431 accelerates muscle regeneration by targeting Pax7 in mice. Therefore, we discuss this report in the text. In that case, miR-431 acted independently of Rtl1 and our data indicate this was not the case in Pat-Rtl1Δ and Mat-Rtl1Δ because the Pax7 level not affected in the absence of miR-431 in Mat-Rtl1Δ and was rather upregulated in Pat-Rtl1Δ, which is inconsistent with their data.

© 2020. Published by The Company of Biologists under the terms of the Creative Commons Attribution License (https://creativecommons.org/licenses/by/4.0/).

#4 Lane 97 I am not sure that I understand correctly the technical importance, if any, of using fixation with SUPER FIX, and its potential link with the fact that Mat-KO mice displayed severe shrinkage Could you please comment ?

We added the data from Elastica Van Gieson (EVG) staining in Supplementary materials Fig. S7A according to the suggestion of reviewer 2. The separation of extracellular matrix (collagen) and muscle fibers, both stained in red, can be clearly seen in the Mat-Rtl1Δ case, indicating the muscle fibers of Mat-Rtl1Δ were detached from the ECM by severe shrinkage. We think this demonstrates that the overexpression of RTL1 actually causes structural changes in the muscle fibers, suggesting this can inhibit the muscle function, thus leading to respiratory problems in Mat-Rtl1Δ neonates. We think this is also related to the etiology of the Kagami-Ogata syndrome.

#5 - Fig.3A Why DAPI staining is shown in red, and not blue as classically used, including in the flanking panel 3B. To further demonstrate the specificity of the detected IF signals, it might also be appropriated to use Pat-KO samples as negative controls.

In the original figure, DAPI was shown in blue and Rtl1 in red. Please check this. The Pat-Rtl1Δ samples were also shown in the original Supplementary materials Fig. S7C (Supplementary materials Fig. S8C in this revised version).

#6 – The lack of Rtl1 is accompanied by increased proliferation rate of satellites cells (SCs). Did the authors test whether the expression of the other flanking imprinted genes remains unaffected in these in vitro cellular models? Optional – Can we envisage performing rescue experiments by stably integrating Rtl1 transgene in SCs prepared from Pat-KO? If possible, the growth of “rescued SCs” could be used as a phenotypic read-out.

Thank you for this comment. We showed that there are no such changes in the muscles in vivo (Fig. S9). This is consistent with our previous data (Kagami et al., 2008), which showed the severity of Kagami-Ogata syndrome correlates well with RTL1 overexpression and not with other DLK1-DIO3 regions. Therefore, we think it reasonable that we focus on Rtl1 and Rtl1as in this work. However, we agree that the expression of the other flanking imprinted genes in in vitro cellular models would provide further information on muscle development because other imprinted genes, since DLK1 and MIRG (the miR-379-410 cluster) have been suggested to be involved. We address this in the first part of the Discussion section.
Concerning the rescue experiment, we first tried to knock down Rtl1 expression in WT SCs using Rtl1 siRNA, OPTI-MEM and Lipofectamine, to see whether this would result in a phenotype similar to Pat-Rtl1Δ. However, the transfection reagent was so toxic that the cells died. Therefore, the introduction of genes into SCs has proven to be very challenging, so we are unable to address this issue at this time.

#7 Throughout the text, the authors employ the term WT. Does it refer to WT littermates? If yes, the authors should then compare, in my view, Pat-KO and Mat-KO to their respective WT littermates. Accordingly, two WT controls should be shown.

This is a very important point. Yes, there are two WT controls in this experiment. As you can see, there were no differences between WT(p) and WT(m) in term of muscle structure and development. We agree that we should show both, so added an image of the WT corresponding to Pat-Rtl1Δ and Mat-Rtl1Δ in Fig. 1B, Fig. 2 and Supplementary materials Fig. S5 and S7. We also presented the strategy for the making of Pat-Rtl1Δ and Mat-Rtl1Δ in Supplementary materials Fig. S2C.

#8 Lane 159 - Title “Temple” and not “Tample”

We changed this to Temple.

#9 Lane 212 Therefore, it is clear that combined and differential involvement of The authors may also include additional references indicating that Dlk1-Dio3-derived miRNAs play some roles in muscular system and, in doing so, may thus contribute to phenotype described in Temple and Kagami-Ogata syndromes. Among others are Wüst et al (2018) Cell Metabol; Castel et al (2018) Sci Rep., Gao et al (2015) PNAS

© 2020. Published by The Company of Biologists under the terms of the Creative Commons Attribution License (https://creativecommons.org/licenses/by/4.0/).

Thank you very much for another important point. We agree that the relationship of the imprinted genes in the DLK1-DIO3 region to muscular hypertrophy, development and regeneration is very important. Therefore, we include the references suggested and discuss this in the first part of the Discussion in detail.

#10 Beside its role in muscle, Rtl1 was previously shown to play some roles in placentation, notably in the maintenance of capillaries. Although I am fully aware that the precise molecular mode of action of Rtl1 is beyond the scope of the present manuscript, can the authors speculate on how Rtl1 may exert distinct functions in two different tissues? Is there any common theme that could explain, at least partly, why Rtl1 appears so crucial for both muscular and placental development? Muscle and placenta-derived cells can differentiate into syncytium. As a case in point, it is well established that retroviral protein co-option (e.g. Syncytin-1) plays prominent roles in Syncytiotrophoblasts. Could it be that altered dosage in Rtl1 impacts indirectly on cell fusion processes? This is obviously pure speculation.

Thank you for your very important comment. That is what we would like to know. However, it is very difficult to answer at the moment. We don't know how it works in placentas nor muscles. We also don't know whether the same function of RTL1 is functional in both cases. Only we have now is an information that REG11(RTL1) has a retroviral type of protease domain. Then, we are trying to make KI mice that has an inactive protease motif. We hope it will really work in both (or either) placenta and muscle cases and give us a que to solve the question. We think it possible that protease action of RLT1 regulates different target proteins in placentas and muscles.

Second decision letter

MS ID#: DEVELOP/2019/185918

MS TITLE: Lack and overexpression of Rtl1 in the mouse cause distinct muscle abnormalities related to the Temple and Kagami-Ogata syndromes, respectively

AUTHORS: Moe Kitazawa, Shinichiro Hayashi, Michihiro Imamura, Yumiko Oishi, Shin'ichi Takeda, Tomoko Kaneko-Ishino, and Fumitoshi Ishino

I have now received all the referees reports on the above revised manuscript, and have reached a decision. The referees' comments are appended below, or you can access them online: please go to BenchPress and click on the 'Manuscripts with Decisions' queue in the Author Area.

The overall evaluation is positive and we would like to publish a revised manuscript in Development, provided that comments raised by Reviewer 2 can be satisfactorily addressed. Please attend to all of the comments raised by Reviewer 2 in your revised manuscript. Please make sure that you submit a revised manuscript with all changes highlighted and submit a detailed point-by-point response that states the exact changes you have made to the revised manuscript. If you do not agree with any of their criticisms or suggestions explain clearly why this is so.

We are aware that you may currently be unable to access the lab to undertake experimental revisions. If it would be helpful, we encourage you to contact us to discuss your revision in greater detail. Please send us a point-by-point response indicating where you are able to address concerns raised (either experimentally or by changes to the text) and where you will not be able to do so within the normal timeframe of a revision. We will then provide further guidance. Please also note that we are happy to extend revision timeframes as necessary.

© 2020. Published by The Company of Biologists under the terms of the Creative Commons Attribution License (https://creativecommons.org/licenses/by/4.0/).

Reviewer 1

Advance summary and potential significance to field

As in my earlier report.

Comments for the author

In their revised manuscript the authors carefully addressed most of the points raised by this reviewer, who has no further suggestions for change.

Reviewer 2

Advance summary and potential significance to field

In the manuscript entitled Â“Lack and overexpression of Rtl1 cause distinct muscle abnormalities related to Temple and Kagami-Ogata syndromes, respectivelyÂ”, Kitazawa and colleagues continue their assessment of loss and overexpression of the imprinted Rtl1 gene. In this manuscript, they specifically examined the role of Rtl1 in muscle cells. They found that paternal Rtl1 deletion and maternal Rtl1 deletion (deletes maternally expressed microRNAs, resulting in Rtl1 overexpression) produced neonatal skeletal muscle defects and abnormalities in satellite cell proliferation. They conclude that deletion of the paternal Peg11 allele and deletion of the maternal Peg11as allele represent models for Temple and Kagami-Ogata syndromes. The authors make contributions to our understanding of Rtl1Â's role in muscle cells.

Comments for the author

The authors addressed the majority of my comments. The authors indicated that those concerning further mechanistic insight was beyond the scope of the current manuscript. The modifications to the manuscript provide additional clarity. There are a few concerns remaining.

1. The authors examine a number of fetal, and neonatal stages. In Figure S3, the authors indicate that P0-P15 is neonatal. Please describe the specific age of fetuses and mice in text and figures to provide further clarity.
2. The new supplemental data in the discussion is out of place and is an overinterpretation. The authors' statement that "We also observed the re-expression of RTL1 in regenerating muscles (Supplementary materials Fig. S11), indicating an involvement of RTL1 in the basic eutherian muscle generation program." Activation of RTL1 expression does not indicate a role in a eutherian muscle generation program. (What are TA muscles?)
3. Discussion is long. The evolutionary discussion is highly speculative.
4. Fig 3, abdominal muscle WT(m) controls are needed.

Minor Comments
1. Line 140 and lines 141-143, are the bar graphs refering to Fig 2D?
2. Line 150, when the authors state "shifted lower" do they mean "smaller size muscle fiber CSA"?
3. Lines189-191, the authors stated regarding Fig 4B, that "where the muscle stem cell marker, Pax7, and early muscle differentiation markers, Myf5 and MyoD, were typically decreased, whereas the levels of late muscle differentiation markers, MCK and Myh4, were increased". This appears to be the overall pattern. The authors did not comment on significant changes in Pat-Rtl1 tissue.

Previous Major Comments
2. We are left with how PEG11, a retrotransposon, actually regulates muscle development and the mechanism through which it acts.

Author: It is a very important question and we really want to know the answer, but it is a very difficult challenge. All that we know at present is that REG11(RTL1) has a retroviral type of protease domain. We are currently engaged in an effort to make KI mice that have an inactive protease motif. We hope this effort will succeed and provide a means to solve the question.

© 2020. Published by The Company of Biologists under the terms of the Creative Commons Attribution License (https://creativecommons.org/licenses/by/4.0/).

Reviewer: The authors specifically discuss how work RTL1 works in muscle fibers in the discussion, and yet they do not state that the mechanism is unknown. At the very least, they authors need to add that the mechanism is unknown and that future studies should be directed to mechanistic biochemical and genetic investigations.

3. Abstract and discussion. The authors present the idea that Peg11 evolved as an adaptation to eutherian viviparous reproduction. This is pure speculation, detracts from the primary story, and lacks experimental data. I strongly suggest that the evolution sections be removed from the manuscript.

We agree with this comment. We deleted the sentence from the abstract and replaced it with the following.
"This is the first example of an LTR retrotransposon-derived gene specific to eutherians contributing to the evolution of eutherian skeletal muscle." Moreover, the most interesting and important point of RTL1 (PEG11) is that it is a eutherian-specific acquired gene, so it should be discussed from the point of view of evolution. In the discussion, we clearly indicate this to be speculation and discuss it as shown below:
In lines 370-375 "We speculate that it also plays a role as a suppressor of DESMIN to prevent fast and vigorous muscle movement in the fetal/neonatal stages by interacting or interfering with DESMIN. This speculation is supported by the fact that Desmin KO mice exhibit no gross abnormality in the fetal/neonatal periods, but rather, exhibit severe muscle defects around 2 weeks after birth (Milner et al., 1996; Li et al.,
1997)." And in lines 379-383, "This view appears to be consistent with the fact that human babies and mouse pups exhibit slow and weak muscle movement just before and after birth. This kind of inhibitory function seems advantageous for both mothers and fetuses/neonates to ensure safety for pregnancy and child-rearing, implying that it was one of important adaptations to the mammalian viviparaous reproduction system."

Reviewer: The statement "This is the first example of an LTR retrotransposon-derived gene specific to eutherians contributing to the evolution of eutherian skeletal muscle" is an overinterpretation as the authors do not perform any studies examining the evolution of eutherian skeletal muscle nor on multiple eutherians.

7. Lines 159-184, this section on evolution should be deleted. While the authors summarize the findings of papers on phenotypes in humans in Table S1-S3, the data was not produced by the authors. This section is also presented in discussion, where it belongs.

Authors: We could not identify the evolutionary statement in Lines 159-184 but reduced the opinion throughout the text. Concerning to the Table S1, the opinion on this point differs among the reviewers. The other two reviewers suggest the data should include Table 1 in the text for a detailed comparison. Therefore we moved the Table S1 to Table 1.

Reviewers: My apologies for not being clear. The section "Rtl1 Pat- and Mat-Rtl1Δ mice are good models in Temple and Kagami-Ogata syndromes, respectively" (lines 214-245) should be in the discussion and not in the results section as it is a comparison of the authors data and the literature.

Second revision

Author response to reviewers' comments

Thank you very much for your suggestions and comments.
We addressed all your concerns and surely hope that you found the changes we made to be satisfactory.

© 2020. Published by The Company of Biologists under the terms of the Creative Commons Attribution License (https://creativecommons.org/licenses/by/4.0/).

Reviewer 2 Comments for the author
The authors addressed the majority of my comments. The authors indicated that those concerning further mechanistic insight was beyond the scope of the current manuscript. The modifications to the manuscript provide additional clarity. There are a few concerns remaining.

1.The authors examine a number of fetal, and neonatal stages. In Figure S3, the authors indicate that P0-P15 is neonatal. Please describe the specific age of fetuses and mice in text and figures to provide further clarity.

We have already explained about specific age (stage) of fetus (embryonic day (d) 8.5, 9.5, 10.5, 12.5, 14.5, 16.5, 18.5) and neonate (Postnatal day (P) 0, 3, 7, 15) in the text (lines 117-120) and figure legend.

2.The new supplemental data in the discussion is out of place and is an overinterpretation. The authors' statement that "We also observed the re-expression of RTL1 in regenerating muscles (Supplementary materials Fig. S11), indicating an involvement of RTL1 in the basic eutherian muscle generation program." Activation of RTL1 expression does not indicate a role in a eutherian muscle generation program. (What are TA muscles?)

For the reviewer's last question, we observed the RTL1 expression in regenerating TA (tibialis anterior) muscles in the Supplementary materials Fig. S11. Concerning the first comment, we discuss the RTL expression in muscle regeneration, here. In the preceded sentence we cited, "Recently, Loo et al. reported that RTL1 is involved in muscle regeneration under the control of the LINC component SUN1." Then, we presented our result as the Supplementary material Fig. S11. Therefore, we think this sentence is situated in the right place. But, we agree on the reviewer's comment "Activation of RTL1 expression does not indicated a role in a eutherian muscle generation program" is an overinterpretation. We changed the sentence below with a statement of limitation of our study according to this reviewer's previous major comment 2 as below (lines 337-341):
We also observed the re-expression of Rtl1 in regenerating muscles, suggesting a possible role of Rtl1 in the muscle regeneration as well as muscle generation in eutherians although the mechanism of RTL1 protein in these processes is unknown and that future studies should be directed to mechanistic biochemical and genetic investigations.

3.Discussion is long. The evolutionary discussion is highly speculative.

According to the reviewer's comment, we total rearranged this part by mostly discussing the putative RTL1 muscle function related to DESMIN from the viewpoint of current eutherian developmental system, not evolution and deleting the sentences on muscle evolution of in the bilaterians. By this change, we only touch the evolutionary issue on the last sentence of this part as below:
Lines 342-374: Our study clearly demonstrates that RTL1 is of critical physiological significance because it plays a major role in the maturation and maintenance of fetal muscle fibers (Figs. 2 and 4), therefore, its loss and overproduction affect the muscle phenotypes of Temple and Kagami-Ogata syndrome, respectively (Table1 and Supplementary material Tables S1-3). Then, how does RTL1 work in the fetal/neonatal muscle fibers? An immunostaining experiment revealed that RTL1 is closely located to DESMIN at the level of the Z-disc (Fig.3). DESMIN is known as one of a sarcomeric cytoskeleton showing some of links between membranes and sarcomeres at the Z-disc, playing as a sub-sarcolemmal protein comprised of costamere, a structural-functional component of the striated muscle cells at the periphery of the Z-disc (Goldfarb and Dalakas, 2009). This muscle-specific complex plays an important role in connecting the force-generating sarcomeres with the sarcolemma, which helps to couple the sarcomere to the extracellular matrix (ECM). Thus, it is likely that the RTL1 protein plays specific role in the function of fetal/neonatal muscle fibers, such as stabilizing the muscle contractile apparatus and/or regulating muscle constriction associated with DESMIN. Therefore, its loss and overproduction affect the strength of muscle fibers as shown in Figs. 2G and 4G. We also speculate that at the normal expression level, the RTL1 protein also plays a role as a suppressor of DESMIN to prevent fast and vigorous muscle movement in the fetal/neonatal stages by interacting or interfering with DESMIN. This speculation is supported by the fact that Desmin KO mice exhibit no gross abnormality in the fetal/neonatal periods, but rather, exhibit severe muscle defects around 2 weeks after birth (Milner et al., 1996; Li et al., 1997) when Peg11/Rtl1 expression ceases in the skeletal muscles (Fig. 1). Thus, it is possible that

© 2020. Published by The Company of Biologists under the terms of the Creative Commons Attribution License (https://creativecommons.org/licenses/by/4.0/).

the DESMIN protein starts functioning at the Z-disc with the disappearance of the RTL1 protein at the Z-disc. In this regard, it is suggestive that no Rtl1 expression is observed in the heart throughout development (Figs. 1A and B), because the cardiac muscles must continuously function after the formation of the heart at d8.5. This view appears to be consistent with the fact that human babies and mouse pups exhibit slow and weak muscle movement just before and after birth. This kind of inhibitory function of RTL1 in the fetal/neonatal muscles seems advantageous for both mothers and fetuses/neonates to ensure safety for pregnancy and child-rearing in the current eutherian reproductive system. Our study also demonstrates that the eutherian skeletal muscle is unique because of the essential recruitment of RTL1 in fetal/neonatal muscle development, implying that it was one of adaptations to the mammalian viviparaous reproduction system.

4.Fig 3, abdominal muscle WT(m) controls are needed.

We added control figure.
But, we apologize that Fig. 3 presented the result of forelimb, not abdominal muscle. It was our mistake and we correct the sample name in the revised version of the manuscript.

Minor Comments
1.Line 140 and lines 141-143, are the bar graphs refering to Fig 2D?

Yes. We indicated bar graphs of Fig. 2D. But we agree that the site of explanation (statistical significance is shown by the * in each bar in Figs. 2x) is not adequate. We deleted it from lines 138-141 and added to line 148.

2.Line 150, when the authors state “shifted lower” do they mean “smaller size muscle fiber CSA”?

Yes. We changed “shifted lower” to “shifted to smaller size” in line 147.

3.Lines189-191, the authors stated regarding Fig 4B, that “where the muscle stem cell marker, Pax7, and early muscle differentiation markers, Myf5 and MyoD, were typically decreased, whereas the levels of late muscle differentiation markers, MCK and Myh4, were increased”. This appears to be the overall pattern. The authors did not comment on significant changes in Pat-Rtl1Δ tissue.

Thank you for your comment. We added a sentence of “Pax7, Myf5, MCK and Myh4 significantly increased in Pat-Rtl1Δ compared to WT, respectively.” in lines 192-193.

Previous Major Comments
2. We are left with how PEG11, a retrotransposon, actually regulates muscle development and the mechanism through which it acts.
Author: It is a very important question and we really want to know the answer, but it is a very difficult challenge. All that we know at present is that REG11(RTL1) has a retroviral type of protease domain. We are currently engaged in an effort to make KI mice that have an inactive protease motif. We hope this effort will succeed and provide a means to solve the question.
Reviewer: The authors specifically discuss how work RTL1 works in muscle fibers in the discussion, and yet, they do not state that the mechanism is unknown. At the very least, they authors need to add that the mechanism is unknown and that future studies should be directed to mechanistic biochemical and genetic investigations.

As I answered in the part of the Major comment 2, we added a sentence of the limitation of our study that “We also observed the re-expression of Rtl1 in regenerating muscles, suggesting some role of Rtl1 in the muscle regeneration as well as muscle generation in eutherians although the mechanism of RTL1 protein in these processes is unknown and that future studies should be directed to mechanistic biochemical and genetic investigations” in lines 337-341.

3. Abstract and discussion. The authors present the idea that Peg11 evolved as an adaptation to eutherian viviparous reproduction. This is pure speculation, detracts from the primary story, and lacks experimental data. I strongly suggest that the evolution sections be removed from the manuscript.
Author: We agree with this comment. We deleted the sentence from the abstract and replaced it with the following. “This is the first example of an LTR retrotransposon-derived gene specific to

© 2020. Published by The Company of Biologists under the terms of the Creative Commons Attribution License (https://creativecommons.org/licenses/by/4.0/).

eutherians contributing to the evolution of eutherian skeletal muscle." Moreover, the most interesting and important point of RTL1 (PEG11) is that it is a eutherian-specific acquired gene, so it should be discussed from the point of view of evolution. In the discussion, we clearly indicate this to be speculation and discuss it as shown below:In lines 370-375 "We speculate that it also plays a role as a suppressor of DESMIN to prevent fast and vigorous muscle movement in the fetal/neonatal stages by interacting or interfering with DESMIN. This speculation is supported by the fact that Desmin KO mice exhibit no gross abnormality in the fetal/neonatal periods, but rather, exhibit severe muscle defects around 2 weeks after birth (Milner et al., 1996; Li et al., 1997)." And in lines 379-383, "This view appears to be consistent with the fact that human babies and mouse pups exhibit slow and weak muscle movement just before and after birth. This kind of inhibitory function seems advantageous for both mothers and fetuses/neonates to ensure safety for pregnancy and child-rearing, implying that it was one of important adaptations to the mammalian viviparaous reproduction system."
Reviewer: The statement "This is the first example of an LTR retrotransposon-derived gene specific to eutherians contributing to the evolution of eutherian skeletal muscle" is an overinterpretation as the authors do not perform any studies examining the evolution of eutherian skeletal muscle nor on multiple eutherians.

There maybe some mistake in the last revised version of manuscript. As you can see in the final PDF version in the WEB site, we have already change this part as below:
"This is the first example of an LTR retrotransposon-derived gene specific to eutherians contributing to eutherian skeletal muscle development".

7. Lines 159-184, this section on evolution should be deleted. While the authors summarize the findings of papers on phenotypes in humans in Table S1-S3, the data was not produced by the authors. This section is also presented in discussion, where it belongs.
Authors: We could not identify the evolutionary statement in Lines 159-184 but reduced the opinion throughout the text. Concerning to the Table S1, the opinion on this point differs among the reviewers. The other two reviewers suggest the data should include Table 1 in the text for a detailed comparison. Therefore, we moved the Table S1 to Table 1.
Reviewers: My apologies for not being clear. The section "Rtl1 Pat- and Mat-Rtl1Δ mice are good models in Temple and Kagami-Ogata syndromes, respectively" (lines 214-245) should be in the discussion and not in the results section as it is a comparison of the authors data and the literature.

According to the Reviewers 1 and 3 suggestions that the previous Table S1 data should be included in the text as Table 1 for a detailed comparison, we moved the Table S1 to Table 1 in the text. We also think this change is better although this part includes the comparison with the literature. We surely hope that the reviewer 2 would understand this change.

Third decision letter

MS ID#: DEVELOP/2019/185918

MS TITLE: Lack and overexpression of Rtl1 in the mouse cause distinct muscle abnormalities related to the Temple and Kagami-Ogata syndromes, respectively

AUTHORS: Moe Kitazawa, Shinichiro Hayashi, Michihiro Imamura, Yumiko Oishi, Shin'ichi Takeda, Tomoko Kaneko-Ishino, and Fumitoshi Ishino
ARTICLE TYPE: Research Article

I am happy to tell you that your manuscript has been accepted for publication in Development, pending our standard ethics checks.

© 2020. Published by The Company of Biologists under the terms of the Creative Commons Attribution License (https://creativecommons.org/licenses/by/4.0/).
